# BI 905711, a TRAILR2/CDH17 Bispecific Antibody, Alone or with Chemotherapy for Patients with Advanced Gastrointestinal Cancers: Phase I Study Findings

**DOI:** 10.1158/2767-9764.CRC-25-0638

**Published:** 2026-05-14

**Authors:** James J. Harding, Ralf Hofheinz, Elena Élez, Yasutoshi Kuboki, Drew W. Rasco, Michael Cecchini, Lin Shen, Min He, Shorena Archuadze, Niraj Chhaya, Franziska Haderk, Lisa Mészáros, Stefan Wölke, Tetsuya Katakabe, Robert M. Lorence, Eric Van Cutsem, Scott Kopetz, Shubham Pant

**Affiliations:** 1 Department of Medicine, Memorial Sloan Kettering Cancer Center, New York, New York.; 2 Weill Cornell Medical College, New York, New York.; 3 Universitätsklinikum Mannheim, Mannheim, Germany.; 4 Vall d’Hebron University Hospital, Barcelona, Spain.; 5 National Cancer Center Hospital East, Kashiwa, Japan.; 6 The START Center for Cancer Research, San Antonio, Texas.; 7 Yale Cancer Center, Yale University, New Haven, Connecticut.; 8 Peking University Cancer Hospital and Institute, Beijing, China.; 9 Boehringer Ingelheim Pharmaceuticals Inc., Ridgefield, Connecticut.; 10 Boehringer Ingelheim Pharma GmbH and Co KG, Biberach an der Riss, Germany.; 11 Nippon Boehringer Ingelheim Co., Ltd., Tokyo, Japan.; 12 University Hospitals Gasthuisberg, University of Leuven (KUL), Leuven, Belgium.; 13 University of Texas MD Anderson Cancer Center, Houston, Texas.

## Abstract

**Purpose:**

BI 905711, a TRAILR2/cadherin-17 (CDH17) bispecific antibody, demonstrated preclinical apoptotic pathway activation and antitumor activity. Two phase Ia/Ib studies tested BI 905711 monotherapy (NCT04137289) or combination therapy (NCT05087992) in advanced, refractory gastrointestinal (GI) cancers.

**Patients and Methods:**

Both studies aimed to determine the maximum tolerated dose (MTD; phase Ia) and recommended phase II dose (RP2D)/recommended dose for expansion (RDE; phase Ib). In phase Ia, patients received BI 905711 monotherapy (0.02–4.8 mg/kg) or 0.6 to 1.2 mg/kg plus biweekly folinic acid (leucovorin), 5-fluorouracil, and irinotecan (FOLFIRI) and bevacizumab. Phase Ib assessed selected doses and regimens given biweekly or weekly (3 weeks on and 1 week off). Safety, efficacy, and pharmacokinetics/pharmacodynamics were evaluated.

**Results:**

In NCT04137289, 110 patients [median age 61 years; 80% with colorectal cancer; median of three prior therapies (range, 1–6)] received monotherapy. No dose-limiting toxicities (DLT) occurred, MTD was not reached, RP2D was not determined, and 48.2% of patients had treatment-related adverse events (TRAE), most commonly nausea (16.4%). In 104 response-evaluable patients, 22.1% achieved stable disease (SD). In NCT05087992, 12 patients with colorectal cancer (median age 54.5 years) received combination treatment. Two patients reported DLTs, MTD was not reached, and the selected RDE was 0.6 mg/kg plus biweekly FOLFIRI and bevacizumab. Most patients (91.7%) had TRAEs, including decreased appetite (33.3%), alanine transaminase increased, aspartate transaminase increased, and diarrhea (25% each). Eleven patients (91.7%) achieved SD. Pharmacokinetic/pharmacodynamic data indicated linear dose exposure and ≥2-fold activation of plasma caspase 3/7.

**Conclusions:**

In heavily pretreated patients with GI tumors, BI 905711 monotherapy or with FOLFIRI plus bevacizumab displayed a manageable safety profile and limited clinical activity.

**Significance:**

Translating preclinical activity of TRAILR2 agonists into the clinic has been hampered by severe hepatotoxicity. BI 905711, a bispecific antibody against TRAILR2 and CDH17, was developed to enhance efficacy and reduce hepatotoxicity. Two phase 1 studies (NCT04137289 and NCT05087992) demonstrated tolerability and minimal hepatotoxicity, dose-proportional pharmacokinetics, and increased markers of target engagement. Antitumor activity was limited. These data show that BI 905711 has reduced TRAIL-related hepatotoxicity in the clinic.

## Introduction

Gastrointestinal (GI) cancers account for nearly one third of the global cancer incidence and are among the top five causes of cancer-related deaths (1, 2). Although standard-of-care systemic treatments for advanced/metastatic, unresectable, microsatellite stable GI cancers typically include chemotherapy, chemoimmunotherapy, precision medicines, and other biologics, treatment outcomes remain unsatisfactory (3, 4). New effective, targeted therapies could address actual unmet clinical needs for patients with unresectable GI cancers (3), especially for colorectal, pancreatic, and gastric cancers, which are associated with a high disease burden and increasing incidence (1, 2).

As cancer cells often evade apoptosis, the targeted reactivation of apoptotic pathways has been explored as a possible therapeutic approach (5). Tumor necrosis factor–related apoptosis-inducing ligand (TRAIL) is a homotrimeric membrane protein that binds to specific cell surface–anchored death receptors, TRAIL-receptor 1 [TRAILR1, also known as death receptor 4 (DR4)] and TRAIL-receptor 2 [TRAILR2, or death receptor 5 (DR5)]. TRAIL binding to its receptors leads to the activation of caspase 8, which subsequently cleaves and activates the effector caspases 3 and 7 to trigger apoptotic cell death (6). Clinical investigation of certain TRAILR agonists has been hampered by their limited antitumor efficacy or significant hepatic toxicity in phase I trials, due to the expression of TRAILR on hepatocytes (7, 8). Therefore, clinical development of new-generation TRAILR agonists has focused on improving their efficacy and tolerability profile (5).

BI 905711 is a tetravalent, immunoglobulin G1, bispecific antibody that cross-links TRAILR2 with the membrane protein cadherin-17 (CDH17), inducing CDH17-dependent TRAILR2 oligomerization. Supplementary Figure S1 shows the molecular structure and mechanism of action of BI 905711 as a symmetric bivalent bispecific antibody (also known as doppelmab; ref. 9). Preclinical analyses showed that BI 905711 induced apoptosis in a wide range of CDH17-positive colorectal cancer cell lines *in vitro* (8). Triggering of extrinsic apoptosis pathways was strictly CDH17-dependent; the absence of CDH17 resulted in a ≥1,000-fold decrease in potency. In addition, BI 905711 led to significant tumor growth inhibition in patient-derived xenograft (PDX) tumor models of colorectal cancer (8). As CDH17 is not expressed in normal human hepatocytes (10), cross-linking to CDH17 may increase the selectivity of BI 905711 for cancer cells in the GI tract, while sparing hepatocytes (6). CDH17 is overexpressed in the plasma membrane of GI adenocarcinomas in general, including colorectal adenocarcinomas (11), and has been described as both an oncogene and a tumor biomarker (12, 13). In PDX models of colorectal cancer, pancreatic ductal adenocarcinoma (PDAC), and other GI cancers, BI 905711 demonstrated significant inhibition of tumor growth, with tumor regression sustained for up to 50 days. The decreases in tumor size were accompanied by the modulation of drug-relevant apoptosis markers [cleaved caspase 3/7 in the plasma and cleaved caspase 3/8 in tumor tissues, as determined via immunohistochemistry (IHC)], suggesting that caspase activation may be a pharmacodynamic marker of BI 905711 activity (6).

This article describes the results of two phase I studies that evaluated BI 905711 as monotherapy or in combination with chemotherapy [folinic acid (leucovorin), 5-fluorouracil, and irinotecan (FOLFIRI)] and bevacizumab in patients with advanced GI cancers.

## Patients and Methods

### Study design and objectives

These two studies were phase Ia/Ib, open-label, multicenter, international (Europe, Asia, and North America) dose-escalation and -expansion trials (Supplementary Fig. S2). Study NCT04137289 examined BI 905711 as monotherapy in patients with advanced GI cancers and was conducted across 18 sites in eight countries (Belgium, China, France, Germany, Japan, South Korea, Spain, and the United States). Phase Ia explored the safety and maximum tolerated dose (MTD) of BI 905711, as well as its pharmacokinetics, pharmacodynamics, and efficacy, to establish a potentially effective dose range for use in phase Ib. In turn, phase Ib evaluated the efficacy and safety of BI 905711 and aimed to determine the recommended phase II dose (RP2D) in four randomized expansion cohorts of patients with colorectal cancer and one planned (but not implemented) expansion cohort of patients with PDAC.

Study NCT05087992 investigated BI 905711 plus FOLFIRI chemotherapy in combination with bevacizumab in patients with advanced, refractory colorectal cancer. This study was conducted across five sites in Belgium, China, France, Japan, and the United States. Phase Ia of this combination study aimed to determine the MTD and the recommended dose for expansion (RDE), pharmacokinetic/pharmacodynamic parameters, safety profile, and preliminary efficacy. Phase Ib assessed the efficacy and safety of BI 905711 combination therapy, with the aim to determine the RP2D.

The studies were conducted in compliance with the Declaration of Helsinki, the International Council for Harmonisation of Technical Requirements for Pharmaceuticals for Human Use Good Clinical Practice guidelines, and all the relevant local regulations. All patients provided written informed consent. Both studies were approved by the local ethics committees at participating centers.

### Patients

The key inclusion criteria for the monotherapy study were as follows: confirmed, advanced/metastatic, unresectable GI cancers; treatment failure with all available conventional therapies; Eastern Cooperative Oncology Group performance status (ECOG PS) of 0 or 1; and for the phase Ib expansion, a diagnosis of colorectal cancer and ≥1 evaluable lesion as per Response Evaluation Criteria in Solid Tumors version 1.1 (RECIST v1.1). The key exclusion criteria included previous systemic anticancer therapy/radiotherapy within specified timeframe from the last dose to the first dose of BI 905711, any concomitant conditions or malignancies, or known GI disorders.

In the combination study, patients had to have histologically or cytologically confirmed, advanced, unresectable, or metastatic colorectal cancer (phase Ia/Ib); ECOG PS 0 to 1; and progressive disease after prior oxaliplatin-based first-line therapy or within 6 months of oxaliplatin-based adjuvant therapy. For phase Ib, ≥1 measurable target lesion (RECIST v1.1) was required.

The key exclusion criteria for the combination study were as follows: any prior irinotecan-based therapy in the metastatic setting; previous systemic anticancer therapy within specified timeframes or radiotherapy within 4 weeks prior to the start of treatment; and any serious concomitant disease or medical condition affecting compliance or considered relevant for the evaluation of efficacy or safety. The full inclusion and exclusion criteria for both studies are provided in Supplementary Table S1A and S1B.

### Study treatment

The BI 905711 starting dose (0.02 mg/kg every 2 weeks) maximum and total exposures (C_max_ and AUC_0–336_) were modeled and compared with *in vivo* exposure in monkeys administered BI 905711 weekly for 6 weeks (a total of seven administrations). As BI 905711 was administered weekly to monkeys and was administered every other week to humans, the monkey AUC_0–168_ was doubled to arrive at an equivalent exposure more than 2 weeks. At the starting dose, human exposure was estimated to be 1,045- and 650-fold below the C_max_ and AUC_0–336_, respectively, in monkeys at the no observed adverse effect level and >3,600-fold below both parameters at the highest nonseverely toxic dose. Therefore, the starting dose was supported by the 6-week repeat-dose toxicity study in cynomolgus monkeys. This dose level was 30-fold below the predicted human therapeutic dose of 0.6 mg/kg every 2 weeks.

An optimal human therapeutic dose was estimated to be 0.6 mg/kg every other week based on the predicted human PK parameters and the efficacious (≥90% tumor growth inhibition) 2-week exposure (AUC_0–14d_) determined in the mouse GP2d and COLO-205 xenograft colorectal cancer models.

To reduce the number of patients potentially exposed to suboptimal doses, the 0.02 mg/kg every 2 weeks and the 0.06 mg/kg every 2 weeks cohorts were single-patient cohorts before enrolling at least four patients each in subsequent cohorts, which included the estimated human therapeutic dose of 0.6 mg/kg every 2 weeks (Supplementary Fig. S2A and S2B).

In phase Ia of the monotherapy study (NCT04137289), patients received BI 905711 intravenously at a starting dose of 0.02 mg/kg every 2 weeks, with a dose range from 0.02 to 4.8 mg/kg (Supplementary Fig. S2A). In phase Ib, patients were randomized to treatment with 0.6, 1.2, or 2.4 mg/kg BI 905711 every 2 weeks, or with 0.6 mg/kg once weekly (3 weeks on and 1 week off; Supplementary Fig. S2B).

In phase Ia of the combination study (NCT05087992), patients received second-line BI 905711 at a starting dose of 0.6 mg/kg (day 3 of each 14-day cycle) plus FOLFIRI and bevacizumab, as follows: irinotecan 180 mg/m^2^ more than 1.5 hours on day 1; leucovorin 400 mg/m^2^ (in Japan: levoleucovorin 200 mg/m^2^) more than 2 hours on day 1; fluorouracil 400 mg/m^2^ bolus on day 1, or 2,400 mg/m^2^ as a continuous infusion for 46 hours starting on day 1; and bevacizumab 5 mg/kg more than 30 minutes on day 1. In phase Ib (dose expansion), patients were randomized 2:1 to receive BI 905711 (at the RDE) plus FOLFIRI and bevacizumab (arm A) or FOLFIRI and bevacizumab only (arm B; Supplementary Fig. S2).

In both studies, BI 905711 was administered until patients experienced disease progression, unacceptable toxicities, or for any other reason requiring treatment discontinuation.

### Study endpoints and assessments

In phase Ia of the monotherapy study (NCT04137289), the primary endpoints were as follows: (i) the MTD and (ii) the number of patients with dose-limiting toxicities (DLT) during the MTD evaluation period. The full DLT criteria are described in Supplementary Table S2A and S2B. The secondary endpoints were pharmacokinetic parameters [C_max_ (maximum measured concentration of BI 905711 in plasma) and AUC_0–336_ (area under the concentration–time curve of BI 905711 in plasma from 0–336 hours)] and objective response rate (ORR), according to RECIST v1.1. In phase Ib, the primary endpoints were ORR and progression-free survival (PFS). The secondary endpoints were pharmacokinetic parameters (C_max_ and AUC_0–336_) during cycles 1 and 3, radiologic tumor shrinkage, disease control, and duration of overall response. The exploratory endpoints (phase Ia/Ib) included activation of caspase 3/7 (as a marker of induction of apoptosis upon treatment with BI 905711) and CDH17 expression (via IHC) in archival or fresh tumor tissue.

In phase Ia of the combination therapy study (NCT05087992), the primary endpoint was the MTD, based on the number of patients with DLTs during the MTD evaluation period. The secondary endpoints were pharmacokinetic parameters: C_max_ and AUC_0–336_. In phase Ib, the primary endpoint was confirmed ORR, as assessed by the investigator based on RECIST v1.1. The secondary endpoints were PFS, duration of response, disease control rate, maximum tumor shrinkage, and pharmacokinetic parameters (C_max_ and AUC_0–336_).

In both studies, the assessments included the following: safety, evaluated through the incidence and severity of adverse events (AE) according to Common Terminology Criteria for Adverse Events version 5.0, DLTs, laboratory parameters, vital signs, and electrocardiograms. AEs of special interest (AESI) were defined as potential severe drug-induced liver injury (DILI), DLTs, infusion-related reactions, and cytokine release syndrome (CRS). Tumor response was assessed via RECIST v1.1 criteria. PFS was defined as the time from the first treatment administration until tumor progression, according to RECIST v1.1 or death from any cause, whichever occurred first. Disease control was defined as complete response (CR), partial response (PR), or stable disease (SD) according to RECIST v1.1. In the combination study, disease control was defined as CR/PR/SD lasting for ≥16 weeks. The duration of overall response was measured from the time that response criteria are first met until the first date of recurrent or progressive disease, according to RECIST v1.1. Radiologic tumor shrinkage was assessed via computed tomography scan or magnetic resonance imaging. Additional methodology for pharmacokinetic and antidrug antibody (ADA) analyses is provided in Supplementary Materials.

### Statistical analyses

In both studies, phase Ia dose escalation was guided via a Bayesian logistic regression model with overdose control that was fitted to the binary toxicity outcomes (DLTs). The number of patients with DLTs in the MTD evaluation period and all other endpoints were analyzed descriptively. During phase Ib, all primary and secondary endpoints were analyzed descriptively.

## Results

### Patients and treatment—monotherapy study

In the monotherapy study, 110 patients received BI 905711 (*n* = 48 in phase Ia and *n* = 62 in phase Ib). In phase Ia, BI 905711 was administered every 2 weeks at 0.02 (*n* = 1), 0.06 (*n* = 1), 0.2 (*n* = 7), 0.6 (*n* = 8), 1.2 (*n* = 8), 2.4 (*n* = 8), 3.6 (*n* = 8), or 4.8 mg/kg (*n* = 7). In phase Ib, BI 905711 was administered every 2 weeks at 0.6 (*n* = 15), 1.2 (*n* = 15), and 2.4 mg/kg (*n* = 16); in addition, 16 patients received 0.6 mg/kg once weekly.

The baseline demographics and disease characteristics are shown in Table 1. Patients had a median age of 61 years, 57.3% were male, and 72.7% were Caucasian. Most patients (80%) had a primary diagnosis of colorectal cancer and had received a median of three prior therapies (range, 1–6). The median number of BI 905711 treatment cycles was 3 (range, 1–20). The median time on-treatment was 1.4 months (range, 0–10.6 months). Most patients (89 patients, 80.9%) were treated for <3 months; seven patients (6.4%) were treated for ≥6 months. The representativeness of the study population to the general population of patients with colorectal cancer is described in Supplementary Table S3.

**Table 1.** Baseline demographics and disease characteristics.

Characteristic	BI 905711 monotherapy (NCT04137289) Phase Ia/Ib Total (*n* = 110)	BI 905711 combination therapy (NCT05087992) Phase Ia/Ib Total (*n* = 12)
Gender, *n* (%)	​	​
Male	63 (57.3)	6 (50)
Female	47 (42.7)	6 (50)
Median age, years (range)	61 (27–81)	54.5 (42–73)
Median weight, kg (range)	73.5 (39–125.6)	72.7 (52–91.2)
Race, *n* (%)	​	​
Asian	16 (14.5)	6 (50)
Black/African American	4 (3.6)	0 (0)
White	80 (72.7)	6 (50)
Other or missing	10 (9.1)	—
Primary diagnosis, *n* (%)	​	​
Colorectal	88 (80)	12 (100)
PDAC	13 (11.8)	—
Biliary tree	3 (2.7)	—
GI tract	3 (2.7)	—
Other	2 (1.8)	—
Stomach	1 (0.9)	—
ECOG PS, *n* (%)	​	​
0	45 (40.9)	6 (50)
1	65 (59.1)	6 (50)
Mean number of locations of metastatic sites, (range)	2.8 (1–6)	2.1 (1–4)
Mean baseline sum of target lesion diameters, mm (range)	110 (11–350)	87.1 (12–273)
Median number of prior anticancer treatments, (range)	3 (1–6)	2 (1–3)
Prior anticancer treatment, *n* (%)	​	​
Chemotherapy	110 (100)	11 (91.7)
Surgery	92 (83.6)	9 (75)
Antibody therapy	56 (50.9)	1 (8.3)
Radiotherapy	35 (31.8)	1 (8.3)
Immunotherapy	27 (24.5)	​
TKI	26 (26.3)	—
Other	27 (24.5)	3 (25)

Abbreviation: TKI, tyrosine kinase inhibitor.

### Patients and treatment—combination study

In the combination study, 13 patients with colorectal cancer received at least one dose of study medications (Table 1); 12 patients received BI 905711 plus FOLFIRI plus bevacizumab, and one patient was randomized to receive FOLFIRI plus bevacizumab only (arm B of phase Ib). In total, nine patients received 0.6 mg/kg BI 905711 plus FOLFIRI plus bevacizumab (seven in phase Ia and two in phase Ib), and three patients received 1.2 mg/kg BI 905711 plus FOLFIRI plus bevacizumab (all in phase Ia).

The median age for the 12 patients treated with BI 905711 was 54.5 years; 50% were female, 50% were Asian, and patients had received a median of two previous anticancer therapies (Table 1). The median duration of treatment with BI 905711 was 4.6 months (range, 1.5–9.7 months); four patients were treated for ≥6 months.

### DLTs, MTD, and safety with BI 905711 monotherapy

In phase Ia, no DLTs were reported at any of the dose levels tested during the MTD evaluation period; the MTD was not reached, and the RP2D was not determined. Two DLT events were reported outside the MTD evaluation period: grade 3 anemia (treatment-related, serious AESI) and grade 3 nausea (treatment-related, not serious AESI).

Most patients (93.6%) experienced at least one AE; the most common AEs were nausea (25.5%), abdominal pain (21.8%), and anemia (20%; Supplementary Table S4). AEs of grades 1, 2, and 3 occurred in 16.4%, 35.5%, and 29.1% of patients, respectively. Treatment-related AEs (TRAE) occurred in 53 patients (48.2%; Table 2). The most common TRAEs were nausea (16.4%), asthenia (9.1%), fatigue (7.3%), and infusion-related reactions (5.5%). All TRAEs were grade 1, 2, or 3 (23.6%, 19.1%, or 5.5%, respectively). Grade 3 AEs were as follows: increased fatigue and aspartate aminotransferase (AST; two patients each) and increased nausea, anemia, and alanine aminotransferase (ALT; one patient each). There were no grade 4/5 TRAEs. TRAEs led to study discontinuation in 14 patients (12.7%); no patient had a TRAE leading to dose reduction.

**Table 2.** Summary of the most frequently reported TRAEs by CTCAE-preferred term for BI 905711 as monotherapy or combination therapy (≥2 patients).

TRAE by preferred term, *n* (%)	BI 905711 monotherapy (NCT04137289) Phase Ia/Ib (*n* = 110)	BI 905711 combination therapy (NCT05087992) Phase Ia/Ib (*n* = 12)
Any TRAE	53 (48.2)	11 (91.7)
Nausea	18 (16.4)	2 (16.7)
Asthenia	10 (9.1)	—
Fatigue	8 (7.3)	—
Infusion-related reaction	6 (5.5)	—
Decreased appetite	5 (4.5)	4 (33.3)
Diarrhea	5 (4.5)	3 (25)
AST increased	5 (4.5)	3 (25)
Vomiting	5 (4.5)	—
ALT increased	4 (3.6)	3 (25)
Pyrexia	4 (3.6)	2 (16.7)
Anemia	3 (2.7)	2 (16.7)
Lipase increased	3 (2.7)	—
Cytokine release syndrome	2 (1.8)	—
Dry mouth	2 (1.8)	—
Headache	2 (1.8)	—
Neutrophil count decreased	—	2 (16.7)
White blood cell count decreased	—	2 (16.7)
Hypokalemia	—	2 (16.7)

Abbreviation: CTCAE, Common Terminology Criteria for Adverse Events.

Overall, 12.7% of patients reported AESIs, including six infusion-related reactions (5.5%) and two CRS events (1.8%; all grade 1); all these AESIs were treatment-related. No potential severe DILI cases were reported.

In total, 37.3% of patients experienced serious AEs (SAE); these required hospitalization or prolongation of hospitalization in 31.8%. The most frequently reported SAE was malignant neoplasm progression (6.4%); abdominal pain, dyspnea, pulmonary embolism, and sepsis were reported in three patients (2.7%) each. Treatment-related SAEs were reported by 4.5% of patients and included CRS in two patients (1.8%; both grade 1 in intensity), grade 3 anemia; grade 3 fatigue, grade 2 cholestasis, and grade 2 decreased appetite (*n* = 1 each). All treatment-related SAEs resolved after treatment discontinuation, except for fatigue and decreased appetite.

### DLTs, MTD, and safety with BI 905711 combination therapy

Two patients in the combination study had DLTs, both in the 1.2-mg/kg group: grade 3 liver disorder (resolved after dose reduction) and grade 3 diarrhea (resolved after treatment). The MTD of BI 905711 was not reached. The RDE for phase Ib was determined as 0.6 mg/kg.

All 12 patients who received BI 905711 combination therapy experienced AEs (Supplementary Table S4). The most frequent AEs were decreased appetite (91.7%), diarrhea, nausea (66.7% each), decreased neutrophil count (58.3%), and decreased white blood cell count (50%). The most commonly reported TRAEs were decreased appetite (33.3%), increased ALT, increased AST, and diarrhea (25% each; Table 2). Grade 3 TRAEs were decreased neutrophil count, stomatitis, DILI, and liver disorder (*n* = 1 each). There were no grade 4 TRAEs. Grade 3 liver-related TRAEs (DILI and liver disorder) all resolved after treatment discontinuation. One patient discontinued treatment due to grade 2 nausea, and two patients had AEs leading to dose reduction (grade 3 liver disorder and grade 3 diarrhea), both recorded as DLTs.

In total, six patients experienced SAEs; most were not treatment-related. Leukopenia was the only grade 4 SAE; there were no grade 5 SAEs. Treatment-related SAEs occurred in two patients: grade 1 pyrexia in one patient and grade 3 DILI, grade 2 enterocolitis, and grade 1 diarrhea in the other patient; all resolved following treatment discontinuation.

### Efficacy

In the monotherapy study, 104 patients had at least one confirmed tumor assessment and were evaluable for response (Table 3). No patient had a confirmed objective response. In total, 23 patients (22.1%) achieved SD. Among these patients, the mean duration of disease control was 5.2 months (range, 1.5–10.8 months), and four patients had SD that lasted ≥6 months. The maximum change in the sum of target lesion diameters is shown in Supplementary Fig. S3.

**Table 3.** Summary of efficacy data for BI 905711 as monotherapy or combination therapy in patients with available data.

​	BI 905711 monotherapy (NCT04137289) Phase Ia/Ib Total (*n* = 104)^a^	BI 905711 combination therapy (NCT05087992) Phase Ia/Ib Total (*n* = 12)
Disease control, *n* (%)	23 (22.1)	8 (66.7)
95% CI	(14.6–31.3)	(34.9–90.1)
Objective response	0 (0)	0 (0)
95% CI	(0–3.5)	(0–26.5)
CR	0 (0)	0 (0)
PR	0 (0)	0 (0)
SD	23 (22.1)	11 (91.7)
<3 months	13 (12.5)	3 (25)
≥3, <6 months	6 (5.8)	5 (41.7)
≥6 months	4 (3.8)	3 (25)
Progressive disease	80 (76.9)	1 (8.3)
Not evaluable	1 (1)	0 (0)
Median PFS (weeks; 95% CI)	7.3 (6.1–7.9)	30.3 (11.7–39.3)

a One patient was not evaluable for response.

The median PFS was 1.7 months [95% confidence interval (CI), 1.4–1.8; Table 3]; 12 patients (11.1%) had a PFS of at least 4 months (PFS4). Of the 13 evaluable patients with PDAC, seven (53.8%) patients had a best overall response of SD, of whom four (30.8%) remained progression-free for ≥4 months. The median PFS in these 13 patients with PDAC was 2.8 months (range, 1.2–5.4 months).

In the combination study, 12 patients were evaluable for response (Table 3). There were no patients with a confirmed OR. In total, 11 of the 12 patients treated with combination therapy (91.7%) achieved SD, which lasted for ≥6 months in three patients (25%). Eight patients (66.7%) had disease control lasting ≥16 weeks; the median duration of disease control was 7.5 months (range, 3.9–10.7 months). The maximum change in the sum of target lesion diameters is shown in Supplementary Fig. S4. In total, 84.6% of all BI 905711-treated patients had a PFS event. The median PFS was 7 months (95% CI, 2.7–9.1 months; Table 3).

### Pharmacokinetics of BI 905711

**Figure 1.**
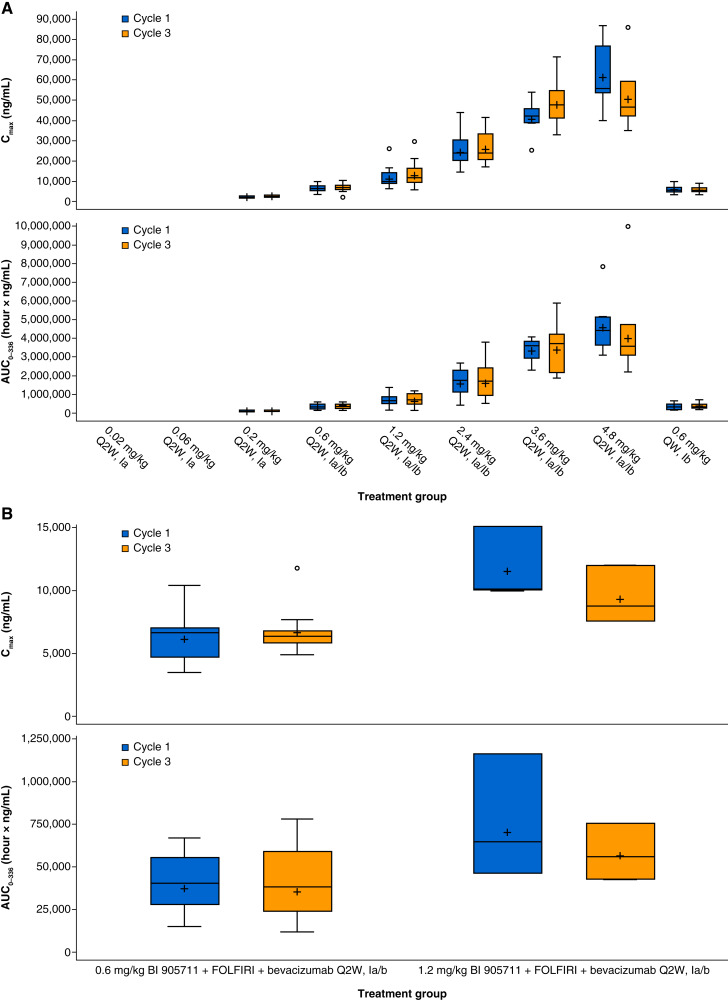
Pharmacokinetic parameters (AUC_0–336_ and C_max_) after infusion of BI 905711 (0.02–4.8 mg/kg) during cycles 1 and 3 of (**A**) monotherapy study (NCT04137289) and (**B**) combination study (NCT05087992). Ia, study phase Ia; Ib, study phase Ib; Q2W, every 2 weeks. For numbers of evaluable patients in cycles 1 and 3, refer to Table 4.

The pharmacokinetic parameters for BI 905711 monotherapy during cycles 1 and 3 are summarized in Table 4 and illustrated in Fig. 1A. In phase Ia, after single (cycle 1) and multiple administration (cycle 3), exposure parameters (C_max_ and AUC_0–336,_) increased with increasing dose. The highest exposure (C_max_ = 61,000 ng/mL and AUC_0–336_ = 4,590,000 h·ng/mL) was observed in the 4.8 mg/kg cohort at cycle 1. No drug accumulation was observed between the first and multiple administrations. Across all cohorts, the geometric coefficient of variation (gCV) of C_max_ and AUC_0-336_ ranged from 16.1% to 76.3%.

**Table 4.** Pharmacokinetic parameters for BI 905711 as monotherapy (NCT04137289)^a^.

Phase Ia																
​	Cycle 1								Cycle 3							
	0.02 mg/kg	0.06 mg/kg	0.2 mg/kg	0.6 mg/kg	1.2 mg/kg	2.4 mg/kg	3.6 mg/kg	4.8 mg/kg	0.02 mg/kg	0.06 mg/kg	0.2 mg/kg	0.6 mg/kg	1.2 mg/kg	2.4 mg/kg	3.6 mg/kg	4.8 mg/kg
C_max_ (ng/mL)																
*N*	1	1	4	8	5	8	8	7	1	1	3	6	4	6	8	6
gMean	306^b^	492^b^	2,110	6,690	11,900	26,200	40,700	61,000	336^b^	497^b^	2,580	7,030	10,300	31,900	47,700	50,300
gCV (%)	—	—	(16.1)	(18.4)	(29.5)	(24.3)	(22.5)	(26.9)	—	—	(17.5)	(20.9)	(44.8)	(26.4)	(23.9)	(32.1)
AUC_0–336_ (h·ng/mL)																
*N*	0	1	4	7	5	8	8	7	0	1	3	6	4	4	7	5
gMean	—	20,000^c^	97,500	391,000	872,000	1,770,000	3,330,000	4,590,000	—	24,500^c^	104,000	358,000	717,000	1,990,000	3,390,000	3,990,000
gCV (%)	—	—	(39.5)	(50.1)	(39.8)	(52)	(22.3)	(29.8)	—	—	(26.8)	(37.2)	(21.3)	(76.3)	(41.2)	(63.3)

Phase Ib								
​	Cycle 1				Cycle 3			
	0.6 mg/kg QW	0.6 mg/kg Q2W	1.2 mg/kg Q2W	2.4 mg/kg Q2W	0.6 mg/kg QW	0.6 mg/kg Q2W	1.2 mg/kg Q2W	2.4 mg/kg Q2W
C_max_ (ng/mL)								
*N*	16	15	15	16	12	10	11	12
gMean	5,740	6,150	10,700	23,100	5,630	6,310	13,600	23,100
gCV (%)	(31)	(31)	(36.7)	(26.6)	(28)	(47.8)	(39.5)	(20.9)
AUC_0–336_ (h·ng/mL)								
*N*	14	15	15	16	10	8	11	11
gMean	323,000^c^	302,000	573,000	1,460,000	354,000^c^	339,000	571,000	1,460,000
gCV (%)	(48)	(40.6)	(55.3)	(50)	(42.9)	(48.1)	(78.9)	(55.9)

Abbreviations: gMean, geometric mean; QW, every week; Q2W, every 2 weeks.

a Pharmacokinetic data were analyzed on the pharmacokinetic analysis set, which included all subjects in the treated set who provided at least one pharmacokinetic endpoint and were not excluded because of a protocol deviation or pharmacokinetic nonevaluability, e.g., patients who did not receive the planned dose (i.e., 50% dose reduction or incorrect dosing).

b For *N* = 1, individual value has been included, instead of gMean.

c The AUC calculation includes an extrapolation from 168 to 336 hours to account for the weekly dosing schedule.

In phase Ib, after first (cycle 1) or multiple administration (cycle 3), C_max_ and AUC_0–336_ also increased with higher doses (Table 4; Fig. 1A). The highest exposure (C_max_ = 23,100 ng/mL and AUC_0–336_ = 1,460,000 h·ng/mL) was observed in the 2.4 mg/kg every 2 weeks cohort at cycles 1 and 3. The gCV of C_max_ and AUC_0–336_ ranged from 20.9% to 78.9%. No drug accumulation was observed between the first and multiple administrations across all dose cohorts.

In phase Ia of the monotherapy study, the proportion of ADA-positive patients was 13% (6/46) at baseline and 32.6% (15/46) after treatment. Likewise, 13.3% (8/60) of patients were ADA-positive at baseline and 41.7% (25/60) after treatment in phase Ib. The number of ADA-positive patients did not increase with higher doses, and there was no indication of an effect of ADA on the pharmacokinetics of BI 905711 (Supplementary Table S5).

As in the monotherapy study, when administered in combination with FOLFIRI and bevacizumab, the exposure of BI 905711 (C_max_ and AUC_0–336_) increased in line with the increasing dose after single or multiple administrations (Table 5; Fig. 1B). The highest exposure (C_max_ = 11,500 ng/mL and AUC_0–336_ = 702,000 h·ng/mL) was observed in the 1.2 mg/kg cohort at cycle 1. No drug accumulation was observed between cycles 1 and 3 in either of the dose cohorts.

**Table 5.** Pharmacokinetic parameters for BI 905711 as combination therapy (NCT05087992)^a^.

Parameter	Phase Ia/Ib	
	Cycle 1	
	0.6 mg/kg	1.2 mg/kg
C_max_ (ng/mL)	​	
*N*	9	3
gMean	6,100	11,500
gCV (%)	(36.6)	(23.9)
AUC_0–336_ (h·ng/mL)	​	
*N*	8	3
gMean	372,000	702,000
gCV (%)	(53.4)	(49.4)

	Cycle 3	
	0.6 mg/kg	1.2 mg/kg
C_max_ (ng/mL)	​	​
*N*	9	3
gMean	6,660	9,280
gCV (%)	(25.4)	(23.8)
AUC_0–336_ (h·ng/mL)	​	​
*N*	8	3
gMean	352,000	565,000
gCV (%)	(73.3)	(29)

Abbreviation: gMean, geometric mean.

a Pharmacokinetic analyses were based on all patients who were treated with BI 905711 combination therapy.

The proportion of ADA-positive patients in the combination study was 25% (3/12) at baseline and 66.7% (8/12) after treatment. There was no indication of any effect of ADA on BI 905711 pharmacokinetics (Supplementary Table S6).

### Pharmacodynamic data for BI 905711

**Figure 2.**
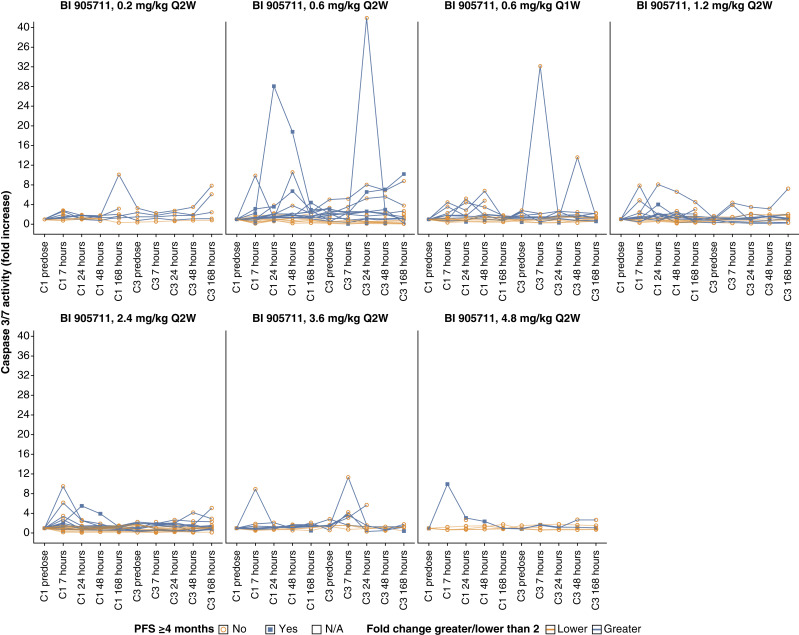
Effect of BI 905711 monotherapy on plasma caspase 3/7 activity in patients with advanced GI cancers (*n* = 104)^a^. *N*/A, not applicable; Q1W, every 1 week; Q2W, every 2 weeks. ^a^The figures show caspase 3/7 activity levels in cycles 1 and 3 for the BI 905711 monotherapy study (NCT04137289). Baseline correction was done to the predose of cycle 1. The blue lines represent patients who showed a ≥2-fold increase in caspase 3/7 activity in either one or both cycles, whereas the orange lines represent patients who did not show a ≥2-fold increase in caspase 3/7 activity. A filled square indicates that the patient successfully achieved PFS of at least 4 months, whereas the open circle indicates a shorter PFS. A lack of symbol indicates missing PFS data.

The activation of caspase 3/7 was examined as an exploratory pharmacodynamic biomarker for induction of apoptosis upon treatment with BI 905711, as was reported in preclinical tumor models (6). Plasma cleaved caspase 3/7 data were available for 104 patients in the monotherapy study and for nine patients in the combination study. In total, 55 patients and one patient in the monotherapy and combination studies, respectively, showed a ≥2-fold plasma caspase 3/7 induction in either cycle 1 or 3 after receiving BI 905711 (Fig. 2). No correlation was observed between caspase 3/7 induction and PFS4 (Fig. 2).

**Figure 3.**
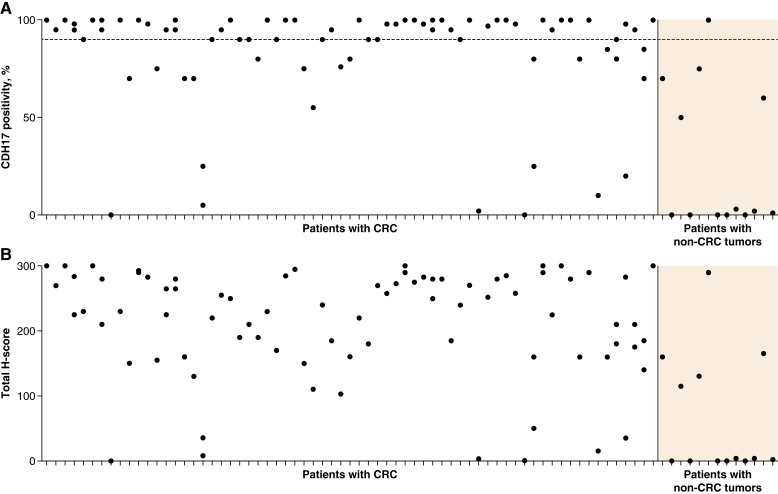
Baseline CDH17 expression levels across colorectal cancer (CRC) and non-CRC tumors in patients treated with BI 905711 monotherapy (NCT04137289 study) as percent positive staining (**A**) and H-score intensity (**B**). Dots represent all biopsies assessed for CDH17 positivity or H-score, including samples from a small number of patients with two biopsies. The dotted line in **A** represents 90% CDH17 positivity by IHC.

In the monotherapy study, high levels of CDH17 expression were observed in patients with colorectal cancer (*n* = 82); the proportion of CDH17-positive cells was 81.8% in PFS4 responders (*n* = 7) and 88% in nonresponders (*n* = 75; Fig. 3A). The mean CDH17 expression at baseline (H-score) for PFS4 responders was 216.1, compared with 226.5 in nonresponders (Fig. 3B). In patients with non–colorectal cancer tumors (*n* = 14), CDH17 expression levels were more heterogeneous, i.e., 25.7% and 25.8% in PFS4 responders (*n* = 3) and nonresponders (*n* = 11), with corresponding H-scores of 44.3 and 66.9, respectively.

## Discussion

These two trials were the first-in-human studies of BI 905711, a TRAILR2 × CDH17 tetravalent bispecific antibody, in patients with advanced GI cancers. In both studies, BI 905711 demonstrated a manageable safety profile but limited antitumor activity.

In the monotherapy study, no DLTs were reported in the MTD evaluation period, and the MTD for BI 905711 was not reached, despite the wide dose range examined (0.02–4.8 mg/kg). A dose of 0.6 mg/kg had previously been predicted to be the human efficacious dose, based on preclinical tumor models and standard pharmacokinetic modeling. Assessment of plasma caspase 3/7 activity in the monotherapy study by dose of BI 905711 administered found that the greatest increase was achieved at a BI 905711 dose of 0.6 mg/kg (Fig. 2). Therefore, 0.6 mg/kg was selected as the lowest dose level for the four dosing cohorts examined in phase Ib to determine the RP2D.

In line with preclinical data (6), there were no DLTs and few liver-related AEs reported with BI 905711 monotherapy, and there were no additional safety concerns reported when using it in conjunction with FOLFIRI and bevacizumab. The MTD was not reached with either schedule, and any liver-related AEs that occurred resolved following treatment discontinuation. These findings contrast with other TRAILR2 agonists, TAS266 and INBRX-109, which have been associated with substantial hepatotoxicity, presumably due to extensive TRAILR2 cross-linking in healthy liver cells (7, 8), whereas TRAILR × CDH17 cross-linking seems to be sparing of liver function after BI 905711 treatment.

In both studies, there was limited antitumor activity with BI 905711. The best overall response was SD in both studies, with only a limited number of patients achieving SD for ≥6 months. Furthermore, the combination therapy regimen did not seem to confer additional efficacy benefits. These results contrasted with prior preclinical data that showed effective *in vitro* antitumor activity for BI 905711 and synergistic effects when administered with irinotecan (6).

Preclinical data had indicated that PDAC was particularly sensitive to BI 905711 activity. Among a pool of PDX PDAC models with sufficient target expression [TRAILR2 >30 transcripts per million (TPM) and CDH17 >20 TPM], antitumor activity was demonstrated in five of eight PDX PDAC models (14). Although no objective responses were observed in BI 905711 monotherapy in patients with PDAC, 4 of 13 patients experienced PFS ≥4 months. This may indicate cytostatic activity or reflect the natural history of the disease in highly selected patients who go on to receive multiple lines of treatment.

The interpretation of SD and PFS4 data in the current study may be complicated, as it is unclear whether the results are due to treatment with BI 905711 or due to the more indolent nature of the patients’ disease.

The observed BI 905711 exposure at the predicted human efficacious dose (0.6 mg/kg) was in line with the predicted pharmacokinetic values from preclinical studies. In both the monotherapy and combination studies, exposure parameters increased linearly with increasing doses. Reassuringly, the individual serum concentration–time profiles of patients in the combination study largely overlapped with the range of pharmacokinetic profiles observed in the corresponding dose groups in the monotherapy study. In addition, both studies showed no drug accumulation between the first and multiple administrations. Furthermore, the number of ADA-positive patients did not increase with higher doses, and there was no indication of an effect of ADA on the pharmacokinetics of BI 905711.

As preclinical tumor models had shown that reductions in tumor size following exposure to BI 905711 were accompanied by the modulation of relevant apoptosis markers (activated caspase 3/7 in plasma and cleaved caspase 3/8 in tumor tissues; ref. 6), the activation of caspase 3/7 was selected here as an exploratory pharmacodynamic biomarker. Although this was an exploratory endpoint, and caspase activation in plasma is not a definitive confirmation of BI 905711 activity, this represents a potential, relatively noninvasive method to monitor BI 905711 target engagement and its correlation with the induction of apoptosis.

Biologically relevant (≥2-fold) changes in plasma levels of activated caspase 3/7 from baseline suggested that the maximal caspase activation occurred at intermediate dose levels of BI 905711 (0.6–1.2 mg/kg every two weeks). However, the extent of caspase 3/7 induction seemed to be limited, and it is currently not possible to predict the minimum level of caspase activation required to induce a clinical response with BI 905711 using this method.

Overall, CDH17 expression levels were high in the majority of tumor samples, as expected for a study in which most patients had colorectal cancer (11). CDH17 expression seemed to be similar in PFS4 responders and nonresponders, suggesting that a potential lack of CDH17 expression in nonresponders may not have been causative of limited antitumor response. Therefore, the therapeutic success of some TRAILR2 agonists might be restricted by an intrinsically high apoptotic threshold in target tumors, as well as rapid cellular adaptation and potentially additional resistance mechanisms.

It is important to note that preclinically with BI905711, there was a bell-shaped dose–response curve *in vitro* in some colorectal cancer cell lines, meaning that at higher concentrations of drug there was less antitumor activity (data on file). Mechanistically, it was hypothesized that concentrations above the optimum may favor individual target recruitment, thus by steric hindrance preventing TRAILR2 cross-linking and therefore reducing efficacy. Importantly, despite the differences in CDH17 and TRAILR2 protein expression levels and the intrinsic sensitivity to TRAILR2 agonists among these cells, the maximal antitumor effect *in vitro* was achieved within a common concentration range of 0.001 to 0.016 nmol/L across the multiple colorectal cancer cell lines. As a result of this effect, the phase 1 clinical program assessed multiple dose expansion cohorts to verify optimal dose. Although no major difference was observed, it is possible that such a reduced activity at higher doses could have impaired observed clinical activity.

In common with other phase I studies, the limitations of these two phase Ia/Ib studies include the small number of patients in some of the treatment cohorts and/or indications. In addition, most patients had received multiple lines of prior therapy (including immunotherapy), and some patients may have been treated with a suboptimal dose of BI 905711, particularly during the dose-escalation phases of these studies. Also, it is possible that the greatest benefit of BI 9057711 may be in combination with chemotherapy, and few patients were finally enrolled in the combination study (NCT05087992).

Based on these preliminary data, the decision was made to terminate the BI 905711 development program due to limited efficacy in this setting, particularly in the context of the evolving treatment landscape for advanced colorectal cancer and other GI cancers. As such, recruitment for the monotherapy study was stopped during the phase Ib expansion part, and no patients with PDAC were enrolled into phase Ib. The recruitment into the combination trial was also prematurely discontinued during the phase Ib expansion part, and no patients with PDAC were enrolled.

### Conclusion

In these two phase I studies, BI 905711 administered as monotherapy or in combination with chemotherapy (±bevacizumab) demonstrated a manageable safety profile and favorable pharmacokinetic properties. However, as limited clinical activity for BI 905711 was observed, with no objective responses across all indications and dose levels (either alone or as part of a combination therapy), the clinical development of BI 905711 was terminated.

## Supplementary Material

Supplementary Materials Supplementary Data

Table S1 Full eligibility criteria for BI 905711 as A) monotherapy (NCT04137289) or B) combination therapy (NCT05087992).

Table S2 Full criteria for DLTs following treatment with BI 905711 as A) monotherapy (NCT04137289) or B) combination therapy (NCT05087992). Any of the following AEs were classified as DLTs, unless unequivocally due to underlying malignancy or an extraneous cause.

Table S3 Representativeness of study participants with advanced CRC.

Table S4 Summary of the most frequently reported adverse events by CTCAE preferred term for BI 905711 as monotherapy (>10% of patients) or combination therapy (≥25% of patients).

Table S5 Individual pharmacokinetic parameters of cycle 3 based on ADA status in monotherapy study.

Table S6 Individual pharmacokinetic parameters of cycle 3 based on ADA status in combination study.

Figure S1 Molecular structure and mechanism of action of BI 905711.

Figure S2 Study design of BI 905711 as A) monotherapy (NCT04137289) or B) combination therapy (NCT05087992).

Figure S3 Maximum percentage change from baseline in the sum of target lesion diameters in study NCT04137289.

Figure S4 Maximum percentage change from baseline in the sum of target lesion diameters in study NCT05087992.

## Data Availability

To ensure independent interpretation of clinical study results and enable authors to fulfill their role and obligations under the ICMJE criteria, Boehringer Ingelheim grants all external authors access to relevant clinical study data. The clinical study data can be made available to qualified researchers upon reasonable request. Researchers should use https://vivli.org/ link to request access to study data and visit https://www.clinicalstudies.boehringer-ingelheim.com/msw/datatransparency for further information.
