# Supplementary material for: BI 905711, a TRAILR2/CDH17 Bispecific Antibody, Alone or with Chemotherapy for Patients with Advanced Gastrointestinal Cancers: Phase I Study Findings
Source: Cancer Res Commun. 2026 May 14;6(5):1123–35. doi: 10.1158/2767-9764.CRC-25-0638 (PMC13172104; doi:10.1158/2767-9764.CRC-25-0638)
Supplement: Supplementary Materials — Supplementary Data [file crc-25-0638_supplementary_materials_suppsd.docx]

**SUPPLEMENTARY MATERIALS**

**Pharmacokinetic and anti-drug antibody (ADA) analyses**

Blood samples for pharmacokinetic and ADA analyses were taken pre-dose at cycles 1, 2, 3, 4, 5, 7, and 13, at end of treatment, and at the 30-day safety follow-up in the monotherapy study, and at cycles 1, 2, 3, 6, 8, 10,12, and 14, at end of treatment, and at the 30-day safety follow-up in the combination study. Furthermore, post-dose pharmacokinetic samples were taken at cycles 1 (after single dose) and 3 (after multiple doses) at 0.5, 7, 24, 48, and 168 hours in both studies. The pre-dose samples of the subsequent cycles were used as the 336-hour timepoint for the Q2W (every 2 weeks) dose regimen.

BI 905711 concentrations were determined by a validated immunoassay.

The presence of ADA was assessed using a validated electrochemiluminescence assay.

**Caspase 3/7 determination**

For analysis of caspase 3/7 in the monotherapy study, longitudinal plasma samples were collected at baseline and after BI 905711 administration (at 7, 24, 48, and 168 hours in cycle 1 and cycle 3).

Caspase 3/7 activation was analyzed via a bioluminescence assay (Caspase-Glo 3/7, Promega). Positive pharmacodynamic changes were pre-defined as ≥2‑fold change from baseline. This preliminary exploratory threshold was defined based on the variability characteristics of caspase 3/7 activity in longitudinal plasma samples from non-treated colorectal cancer (CRC) patients, where most samples show ≤1.99-fold fluctuation over time.

**Cadherin-17 assay’**

Cadherin 17 (CDH17) protein expression in formalin-fixed, paraffin-embedded tumor samples was measured via immunohistochemistry using the anti-CDH17 antibody clone SP183 (Ventana Medical Systems/Roche Diagnostics/Cell Marque). CDH17 expression levels were retrospectively correlated with clinical outcome [i.e., the proportion of patients with progression-free survival (PFS) of at least 4 months (PFS4)].
