# Supplementary material for: BI 905711, a TRAILR2/CDH17 Bispecific Antibody, Alone or with Chemotherapy for Patients with Advanced Gastrointestinal Cancers: Phase I Study Findings
Source: Cancer Res Commun. 2026 May 14;6(5):1123–35. doi: 10.1158/2767-9764.CRC-25-0638 (PMC13172104; doi:10.1158/2767-9764.CRC-25-0638)
Supplement: Table S1 — Full eligibility criteria for BI 905711 as A) monotherapy (NCT04137289) or B) combination therapy (NCT05087992). [file crc-25-0638_table_s1_suppst1.docx]

**Table S1.** Full eligibility criteria for BI 905711 as A) monotherapy (NCT04137289) or B) combination therapy (NCT05087992).

| **A) Study NCT04137289 (BI 905711 monotherapy)** | |
| --- | --- |
| **Inclusion criteria** | 1. Histologically or cytologically confirmed, advanced unresectable or metastatic GI cancers of following histologies:  - Colorectal adenocarcinoma - Gastric adenocarcinoma - Esophageal adenocarcinoma - Pancreatic adenocarcinoma - Cholangiocarcinoma and gallbladder carcinoma.  1. Patients who have failed all available conventional therapies known to confer clinical benefit for their disease based on local approved standards. For patients with CRC, prior treatment with regorafenib or TAS-102 is optional. 2. Phase Ia (dose escalation) only: patient with either measurable or non-measurable/non-evaluable disease. Phase Ia (expanded cohort) and phase Ib (expansion phase) only: at least one target lesion that can be accurately measured per RECIST v1.1. 3. Availability and willingness to undergo tumor biopsy before treatment to provide tumor tissue. Pre-treatment fresh tumor biopsy collections for biomarker analyses are considered optional in phase Ia and mandatory in phase Ib. However, fresh tumor biopsies will not be considered if significant risk procedures are required including (but not limited to) biopsies of the pancreas, or endoscopic procedures extending beyond the esophagus, stomach, or bowel. Only non-significant risk procedures per the investigator’s judgment will be used to obtain any biopsies specified in this study. In case a fresh tumor biopsy cannot be obtained due to before-mentioned reasons, an archived tumor tissue specimen needs to be submitted. 4. Adequate hepatic, renal, and bone marrow functions, as defined by all of the below: 5. Total bilirubin ≤1.5 x institutional ULN (≤3 x institutional ULN for patients with Gilbert’s syndrome) 6. ALT and AST ≤2.5 x institutional ULN (≤5 x institutional ULN for patients with known liver metastases) 7. Serum creatinine ≤1.5 x institutional ULN. If creatinine is >1.5 x ULN, patient is eligible if concurrent creatinine clearance ≥50 ml/min (measured or calculated by CKD-EPI formula or Japanese version of CKD-EPI formula for Japanese patients) 8. ANC ≥1.0 x 10^9^/L 9. Platelets ≥100 x 10^9^/ L 10. Hb ≥9.0 g/dL (without transfusion within previous week) 11. Serum lipase ≤ 1.5 x institutional ULN. 12. Recovery from any AEs according to CTCAE v5.0 of previous anti-cancer therapies to baseline or CTCAE grade 1, except for alopecia CTCAE grade 2, sensory peripheral neuropathy CTCAE grade ≤2 or considered not clinically significant. 13. ECOG performance status ≤1. 14. Life expectancy ≥3 months in the opinion of the investigator. 15. Of legal adult age (according to local legislation) at screening. 16. Signed and dated written informed consent in accordance with ICH-GCP and local legislation prior to admission to the trial. 17. Male or female patients. WOCBP and men able to father a child must be ready and able to use highly effective methods of birth control per ICH M3 (R2) that result in a low failure rate of less than 1% per year when used consistently and correctly. |
| **Exclusion criteria** | - 1. Treatment with a systemic anti-cancer therapy or investigational drug within 14 days or five half-lives (whichever is shorter) of the first treatment with the study medication.  1. Radiation therapy with extensive large field involving parenchymal organs in chest, abdomen, or pelvis within 3 weeks of the first treatment in the study. There is no restriction for minor small field radiotherapy. 2. Any serious concomitant disease or medical condition affecting compliance with trial requirements or which are considered relevant for the evaluation of the efficacy or safety of the trial drug, such as neurologic, psychiatric, infectious disease, or active ulcers (GI tract, skin), or laboratory abnormality that may increase the risk associated with trial participation or trial drug administration, and in the judgment of the investigator, would make the patient inappropriate for entry into the trial. 3. Known pathological condition of the GI tract, liver, and pancreas, excluding the disease under study, that may interfere with assessment of drug safety or may increase the risk of toxicity: 4. Inflammatory bowel disease 5. Chronic pancreatitis 6. Other serious GI pathological conditions by judgment of the investigator e.g., autoimmune disease with GI involvement, unexplained active diarrhea CTCAE grade ≥2, according to CTCAE v5.0. 7. Known history of HIV infection. 8. Any of the following laboratory evidence of hepatitis virus infection. Test results obtained in routine diagnostics are acceptable if done within 14 days before the informed consent date:  - Positive results of HBs antigen - Presence of HBc antibody together with HBV-DNA - Presence of hepatitis C RNA.  1. Active concomitant malignancies, other than the one treated in this trial. 2. Chronic alcohol or drug abuse or any condition that, in the investigator’s opinion, makes the patient an unreliable trial participant or unlikely to comply with the protocol requirements or not expected to complete the trial as scheduled. 3. Women who are pregnant, nursing, or who plan to become pregnant while in the trial; female patients who do not agree to the interruption of breast feeding from the start of study treatment to within 30 days after the last study treatment. 4. Presence of uncontrolled or symptomatic brain or subdural metastases. Inclusion of patients with brain metastases who have completed local therapy and are considered stable by the investigator, or with newly identified asymptomatic brain metastases at screening will be allowed. Use of corticosteroids is allowed if the dose was stable for at least 1 week before the baseline MRI. 5. Patients who are under judicial protection and patients who are legally institutionalized. 6. Major surgery (major according to the investigator’s assessment) performed within 3 weeks prior to treatment start, or planned within 3 months after screening, e.g., hip replacement. 7. Any of the following cardiac criteria: 8. Resting corrected QT interval (QTc) >470 msec 9. Any clinically important abnormalities (as assessed by the investigator) in rhythm, conduction, or morphology of resting ECGs, e.g., complete left bundle branch block, third degree heart block 10. Patients with an EF <50% or the lower limit of normal of the institutional standard will be excluded. Only in cases where the investigator (or the treating physician or both) suspects cardiac disease with negative effect on the EF, will the EF be measured during screening using an appropriate method according to local standards to confirm eligibility (e.g., echocardiogram, multi-gated acquisition scan). A historic measurement of EF no older than 6 months prior to first administration of study drug can be accepted provided that there is clinical evidence that the EF value has not worsened since this measurement in the opinion of the investigator or of the treating physician or both. 11. Known hypersensitivity to the trial medication and/or its components i.e., polysorbate 20, sodium citrate, lysine hydrochloride, sucrose, citric acid. |

| **B) Study NCT05087992 (BI 905711 combination therapy)** | |
| --- | --- |
| **Inclusion criteria** | Applicable to both phase Ia and phase Ib cohorts:   - 1. Signed and dated written informed consent in accordance with ICH-GCP and local legislation prior to admission to the trial.   2. Of legal adult age (according to local legislation) at screening.   3. Histologically or cytologically confirmed, advanced unresectable, or metastatic colorectal adenocarcinoma.   4. ECOG performance status ≤ 1.   5. Life expectancy ≥3 months in the opinion of the investigator.   6. Availability and willingness to provide tumor tissue (fresh biopsy and archival) for biomarker analysis. Only non-significant risk procedures per the investigator’s judgment will be used to obtain any biopsies specified in this study. In case a fresh tumor biopsy cannot be obtained, the recruitment of the patient may proceed on a case-by-case basis after agreement between the investigator and Boehringer Ingelheim. In such a case, an archived tumor tissue specimen must be submitted.   7. Adequate hepatic, pancreatic, renal, and bone marrow functions as defined by all of the below: - Total bilirubin ≤1.5 x institutional ULN - ALT and AST ≤2.5 x institutional ULN or ≤5 x institutional ULN for patients with known liver metastases - Serum creatinine ≤1.5 x institutional ULN. If creatinine is >1.5 x ULN, patient is eligible if concurrent creatinine clearance ≥50 ml/min (≥ 0.05 L/min), measured or calculated by CKD-EPI formula or Japanese version of CKD-EPI formula for Japanese patients - ANC ≥1.5 x 10^9^/L, ≥1.5 x 10^3^/μL, or ≥1500/mm^3^ - Platelets ≥100 x 10^9^/L, ≥100 x 10^3^/μL, or ≥100 x 10^3^/mm^3^ - Hb ≥8.5 g/dl, ≥85 g/L, or ≥5.3 mmol/L (without transfusion within the previous week) - Serum lipase ≤1.5 institutional ULN.   1. Recovery from any AEs of previous anticancer therapies to CTCAE v5.0 grade 1, except for CTCAE grade 2 alopecia or peripheral sensory neuropathy, or other CTCAE grade 2 AEs considered not clinically significant in the investigator’s opinion.   2. Male or female patients. WOCBP and men able to father a child must be ready and able to use highly effective methods of birth control per ICH M3 (R2) that result in a low failure rate of less than 1% per year when used consistently and correctly.   Additionally, criterion 10 is applicable to phase Ia cohort only:   - 1. Patient with either measurable or non-measurable disease.   Additionally, criterion 11 is applicable to phase Ib cohorts only:   - 1. At least one target lesion that can be accurately measured per RECIST v1.1.   2. Histologically or cytologically confirmed, advanced, unresectable, or metastatic, CDH17-positive pancreatic adenocarcinoma.   3. Patients must also meet the following: - CRC expansion cohort: patients who have PD after prior oxaliplatin-based first-line therapy or within 6 months after the end of oxaliplatin-based adjuvant therapy - PDAC expansion cohort: patients who have PD after prior platin and/or gemcitabine-based first line therapy. |
| **Exclusion criteria** | Applicable to both phase Ia and phase Ib cohorts:   1. Any prior irinotecan-based therapy in the metastatic setting. 2. Previous systemic anticancer therapy within the specified timeframe from the last dose intake to the first dose of trial treatment as follows:  - Any non-investigational drug, including anti-angiogenic agents (bevacizumab or ramucirumab or aflibercept) and anti-EGFR antibodies (cetuximab or panitumumab), within 14 days - Any investigational drug or other antibodies including immune checkpoint inhibitors, within 28 days.  1. Currently enrolled in another investigational device or drug trial. Patients who are in follow-up/observation for another clinical trial are eligible. 2. Radiation therapy within 4 weeks prior to start of treatment. However, palliative radiotherapy for symptomatic metastasis is allowed if completed within 2 weeks prior to start of treatment. 3. Any serious concomitant disease or medical condition affecting compliance with trial requirements, or which are considered relevant for the evaluation of the efficacy or safety of the trial drug, such as neurologic, psychiatric, infectious disease, or active ulcers (GI tract, skin), or laboratory abnormality that may increase the risk associated with trial participation or trial drug administration, and in the judgment of the investigator, would make the patient inappropriate for entry into the trial. 4. Known pathological condition of GI tract, liver, and pancreas, excluding the disease under study, that may interfere with assessment of drug safety or may increase the risk of toxicity: 5. Inflammatory bowel disease 6. Chronic pancreatitis 7. Other serious GI pathological conditions by judgment of the investigator e.g., autoimmune disease with GI involvement, unexplained active diarrhea CTCAE v5.0 grade ≥2. 8. Known history of HIV infection. 9. Any of the following laboratory evidence of hepatitis virus infection. Test results obtained in routine diagnostics are acceptable if done within 14 days before the informed consent date:  - Positive results of HBs antigen - Presence of HBc antibody together with HBV-DNA - Presence of hepatitis C RNA.  1. Previous or concomitant malignancies, other than the one treated in this trial within the last 2 years, with exception of the following:  - Effectively treated non-melanoma skin cancers - Effectively treated carcinoma in situ of the cervix - Effectively treated ductal carcinoma in situ - Other effectively treated malignancy that is considered cured by local treatment.  1. Chronic alcohol or drug abuse or any condition that, in the investigator’s opinion, makes the patient an unreliable trial participant or unlikely to comply with the protocol requirements or not expected to complete the trial as scheduled. 2. Women who are pregnant, nursing, or who plan to become pregnant while in the trial; female patients who do not agree to the interruption of breast feeding from the start of study treatment through 6 months after the last study treatment. 3. Presence of uncontrolled or symptomatic brain or subdural metastases. Inclusion of patients with brain metastases who have completed local therapy and are considered stable by the investigator, or with newly identified asymptomatic brain metastases at screening will be allowed. Use of corticosteroids is allowed if the dose was stable for at least 1 week before the baseline MRI. 4. Patients who are under judicial protection and patients who are legally institutionalized. 5. Major surgery (major according to the investigator’s assessment) performed within 28 days prior to treatment start, or planned within 3 months after screening, e.g., hip replacement. 6. Any of the following cardiac criteria: 7. Resting corrected QT interval (QTc) >470 msec based on local assessment 8. Any clinically important abnormalities (as assessed by the investigator) in rhythm, conduction, or morphology of resting ECGs, e.g., complete left bundle branch block, third degree heart block 9. Patients with an EF <50% or the lower limit of normal of the institutional standard will be excluded. Only in cases where the investigator (or the treating physician or both) suspects cardiac disease with negative effect on the EF, will the EF be measured during screening using an appropriate method according to local standards to confirm eligibility (e.g., echocardiogram, multi-gated acquisition scan). A historic measurement of EF no older than 6 months prior to first administration of study drug can be accepted provided that there is clinical evidence that the EF value has not worsened since this measurement in the opinion of the investigator or of the treating physician or both 10. Patients with a history of stroke or myocardial infarction within 6 months prior to screening are not permitted. 11. Known hypersensitivity to the trial medications or their excipients. 12. The patient has any known history or clinical evidence of Gilbert’s Syndrome, or is known to have any of the following genotypes: UGT1A1*6/*6, UGT1A1*28/*28, or UGT1A1*6/*28.   18. Any contradictions to the proposed background therapy, according to the current approved local label. |

Abbreviations: AE, adverse events; ALT, alanine aminotransferase; ANC, absolute neutrophil count; AST, aspartate transaminase; CDH17, cadherin 17; CKD-EPI, Chronic Kidney Disease Epidemiology Collaboration; CRC, colorectal cancer; CTCAE, Common Terminology Criteria for Adverse Events; ECG, electrocardiogram; ECOG, Eastern Cooperative Oncology Group; EF, ejection fraction; EGFR, epidermal growth factor receptor; GI, gastrointestinal; Hb, hemoglobin; HBc, hepatitis B core antibody; HBs, hepatitis B surface antigen; HBV-DNA, hepatitis B virus deoxyribonucleic acid; HIV, human immunodeficiency virus; ICH-GCP, International Council on Harmonization Good Clinical Practice; ICH M3 (R2), ICH guideline M3 (R2) on non-clinical safety studies for the conduct of human clinical trials for pharmaceuticals; MRI, magnetic resonance imaging; PD, progressive disease; PDAC, pancreatic ductal adenocarcinoma; QTc, corrected QT interval; RECIST, Response Evaluation Criteria in Solid Tumors; RNA, ribonucleic acid; UGT1A1, uridine diphosphate glucuronosyltransferase 1-1; ULN, upper limit of normal; WOCBP, women of childbearing potential.
