# Supplementary material for: BI 905711, a TRAILR2/CDH17 Bispecific Antibody, Alone or with Chemotherapy for Patients with Advanced Gastrointestinal Cancers: Phase I Study Findings
Source: Cancer Res Commun. 2026 May 14;6(5):1123–35. doi: 10.1158/2767-9764.CRC-25-0638 (PMC13172104; doi:10.1158/2767-9764.CRC-25-0638)
Supplement: Table S2 — Full criteria for DLTs following treatment with BI 905711 as A) monotherapy (NCT04137289) or B) combination therapy (NCT05087992). Any of the following AEs were classified as DLTs, unless unequivocally due to underlying malignancy or an extraneous cause. [file crc-25-0638_table_s2_suppst2.docx]

**Table S2.** Full criteria for DLTs following treatment with BI 905711 as A) monotherapy (NCT04137289) or B) combination therapy (NCT05087992). Any of the following AEs were classified as DLTs, unless unequivocally due to underlying malignancy or an extraneous cause.

| 1. **Study NCT04137289 (BI 905711 monotherapy)** | |
| --- | --- |
| **Category** | **Criteria and CTCAE grade defining a DLT** |
| Hematologic  laboratory | - Grade 4 neutropenia lasting ˃7 days. - Grade ≥3 neutropenia with documented infection. - Grade ≥3 febrile neutropenia defined as ANC <1000/mm^3^ and a single temperature of ≥38.3ºC (101ºF) or a sustained temperature of ≥38ºC (100.4ºF) for more than 1 hour; or where there are life-threatening consequences or urgent intervention indicated. - Grade 3 thrombocytopenia (platelet count ≥25,000/m^3^ and <50,000/m^3^) associated with bleeding, excluding grade 1 epistaxis. - Grade 4 thrombocytopenia (platelet count <25,000/m^3^). - Thrombocytopenia or anemia requiring transfusion per local or international guidelines. - Neutropenia that requires administration of hematopoietic growth factor agents per local or international guidelines. |
| Non-hematologic  laboratory | - Any grade 3 or grade 4 non-hematologic laboratory value if:   - Medical intervention is required to treat the patient, or   - The abnormality is a serious AE, or   - The abnormality persists >1 week, and considered significant enough to be qualified as DLT in the investigator’s opinion, and confirmed by the SMC. - An elevated AST or ALT value ≥3 x the ULN and an elevated total bilirubin value ≥2 x ULN measured in the same blood draw sample and, at the same time, an alkaline phosphatase value <2 x ULN, as determined by way of protocol-specified lab testing or unscheduled lab testing. - An elevated AST or ALT value ≥5 x ULN and an elevated total bilirubin value  ≥2 x ULN measured in the same blood draw sample, with the exclusion of causes due to underlying diseases (for patients with elevated liver enzymes at baseline). |
| Non-laboratory | - Any grade 4 non-laboratory toxicity possibly related to study therapy, irrespective of whether patient received maximal supportive therapy. - Any grade 3 non-laboratory toxicities despite the use of adequate/maximal medical interventions and/or prophylaxis as dictated by local institutional clinical practices or the judgment of the investigator, except for:   - Fatigue/asthenia present at baseline that worsens on study and lasts less than 7 days   - New onset of grade 3 nausea or grade 3 vomiting lasting ≤48 hours, and which resolved to ≤ grade 1 either spontaneously or with conventional medical intervention   - Nausea or vomiting present at baseline that worsens on-study, and resolves with treatment within 24 hours   - Grade 3 diarrhea not requiring hospitalization, lasting ≤48 hours, and which resolved to ≤ grade 1 either spontaneously or with conventional medical intervention. - Any other toxicity considered significant enough to be qualified as DLT in the opinion of the investigator, and confirmed by the SMC, will be reported as a DLT. - Any toxicity grade ≥2 leading to dose reduction will be considered as a DLT. - Any death not clearly due to the underlying disease or extraneous causes. |
| Treatment delay | - Any toxicity that results in a treatment delay >14 days. |
| 1. **Study NCT05087992 (BI 905711 combination therapy)** | |
| **Category** | **Criteria and CTCAE grade defining a DLT** |
| Treatment delay* | - Any toxicity that results in a treatment delay >14 days. |
| Hematologic  laboratory* | - Grade 4 neutropenia lasting ˃7 days. - Grade ≥3 neutropenia with documented infection. - Grade ≥3 febrile neutropenia defined as ANC <1000/mm^3^ (<1.0 x 10^9^/L, <1.0 x 10^3^/μL) and a single temperature of ≥38.3ºC (101ºF) or a sustained temperature of ≥38ºC (100.4ºF) for more than 1 hour; or where there are life-threatening consequences or urgent intervention indicated. - Grade 3 thrombocytopenia [platelet count <50,000/m^3^ (<50 x 10^9^/L, <50 x 10^3^/μL)] associated with bleeding, excluding grade 1 epistaxis. - Grade 4 thrombocytopenia [platelet count <25,000/m^3^ (<25 x 10^9^/L, <25 x 10^3^/μL)]. - Thrombocytopenia or anemia requiring transfusion per local or international guidelines. - Neutropenia that requires administration of hematopoietic growth factor agents per local or international guidelines. |
| Non-hematologic  laboratory | - Any grade 3 or grade 4 non-hematologic laboratory value if:   - Medical intervention is required to treat the patient, or   - The abnormality is a serious AE, or   - The abnormality persists >1 week, and considered significant enough to be qualified as DLT in the investigator’s opinion, and confirmed by the DRC. - An elevated AST or ALT value ≥3 x the ULN and an elevated total bilirubin value ≥2 x ULN measured in the same blood draw sample and, at the same time, an alkaline phosphatase value <2 x ULN, as determined by way of protocol-specified lab testing or unscheduled lab testing. - An elevated AST or ALT value ≥5 x ULN and an elevated total bilirubin value ≥2 x ULN measured in the same blood draw sample, with the exclusion of causes due to underlying diseases (for patients with elevated liver enzymes at baseline). - Any grade 4 non-laboratory toxicity possibly related to study therapy, irrespective of whether patient received maximal supportive therapy. - Any grade 3 non-laboratory toxicities despite the use of adequate/maximal medical interventions and/or prophylaxis as dictated by local institutional clinical practices or the judgment of the investigator, except for:   - Fatigue/asthenia present at baseline that worsens on study and lasts fewer than 7 days   - New onset of grade 3 nausea or grade 3 vomiting lasting ≤48 hours, and which resolved to grade ≤1, either spontaneously or with conventional medical intervention   - Nausea or vomiting present at baseline that worsens on-study, and resolves with treatment within 24 hours   - Grade 3 diarrhea not requiring hospitalization, lasting ≤48 hours, and which resolved to grade ≤1, either spontaneously or with conventional medical intervention. - Any other toxicity considered significant enough to be qualified as DLT in the opinion of the investigator, and confirmed by the DRC, will be reported as a DLT. - Any toxicity grade ≥ 2 leading to dose reduction will be considered as a DLT. - Any death not clearly due to the underlying disease or extraneous causes. |

*Except if the event is considered due to background therapy only.

Abbreviations: AE, adverse events; ALT, alanine aminotransferase; ANC, absolute neutrophil count; AST, aspartate transaminase; CTCAE, Common Terminology Criteria for Adverse Events; DRC, Data Review Committee; DLT, dose-limiting toxicity; SMC, safety monitoring committee; ULN, upper limit of normal.

**Table S3.** Representativeness of study participants with advanced CRC.

| **Patient populations** | Pooled manuscript population from two Phase Ia/Ib studies | General population: advanced, refractory gastrointestinal cancers |
| --- | --- | --- |
| **Considerations related to:** |  |  |
| Sex | Of the 122 patients assessed, 57% were male and 43% were female. | Overall, CRC incidence is ~32% higher in men than in women overall (40.5 vs. 30.7 cases per 100,000), although differences in incidence between sexes can vary by the patient age group and location of the tumor.^1,2^ |
| Age | Median ages were 61.0 and 54.5 years in the two studies. | According to the US National Cancer Institute, the median age of patients at diagnosis of CRC is ~67 years for colorectal cancer.^3^ This aligns with the American Cancer Society age-distribution data, which shows that 56% of new CRC cases occur at age ≥65 years, 32% at age 50–64 years, and 13% under age 50 years.^2^ |
| Race/ethnicity | Of the 122 patients enrolled across the two studies, 70.5% were White, 18% were Asian, 3.3% were Black or African-American, and 8.2% had other or missing information on race. | CRC incidence is highest in American Indian/Alaska Native and Black populations and is lowest in Asian/Pacific Islander populations. Multiple peer-reviewed analyses confirm this pattern, noting that Black and American Indian populations consistently experience the highest incidence.^1,4^ |
| Geography | These two studies recruited patients from sites in the following countries: Belgium, China, France, Germany, Japan, South Korea, Spain, and USA. | In the USA, overall CRC incidence is 35.3 per 100,000 people and is the leading cause of cancer death in adults aged <50 years.^1^ Outside the USA in 2020, CRC incidence rates were highest in Australia/ New Zealand and European regions (40.6 per 100 000, males) and lowest in several African regions and Southern Asia (4.4 per 100 000, females).^5^ |
| Overall representativeness of this study | In terms of sex and age or participants, the current analysis reflects the CRC incidence data shown in the wider literature. The overall racial composition of the study populations is reflective of that of the general populations of the countries that patients were recruited from and may be further influenced by the requirement to meet study inclusion criteria. Therefore, the racial composition of the current analysis does not accurately reflect the real-world racial subgroups that are predisposed to develop CRC. | |

CRC, colorectal cancer

1. Siegel RL, *et al*. Colorectal cancer statistics. CA: A Cancer Journal for Clinicians. 2026;76: https://doi.org/10.3322/caac.70067.
2. American Cancer Society. Colorectal Cancer Facts & Figures 2023-2025. Atlanta: American Cancer Society; 2023. Available online <https://www.cancer.org/content/dam/cancer-org/research/cancer-facts-and-statistics/colorectal-cancer-facts-and-figures/colorectal-cancer-facts-and-figures-2023.pdf> accessed March 2026.
3. National Cancer Institute. Cancer Causes and Prevention. <https://seer.cancer.gov/statfacts/html/all.html>, accessed Mach 2026.
4. Pankratz VS, et al. Cancer Incidence Trends Across Regions of the United States From 2001 to 2020– A United States Cancer Statistics Analysis. Cancer Control. 2024; 10732748241300653.
5. Morgan E, et al. Global burden of colorectal cancer in 2020 and 2040: incidence and mortality estimates from GLOBOCAN Gut 2023;72:338-344.
