# Supplementary material for: BI 905711, a TRAILR2/CDH17 Bispecific Antibody, Alone or with Chemotherapy for Patients with Advanced Gastrointestinal Cancers: Phase I Study Findings
Source: Cancer Res Commun. 2026 May 14;6(5):1123–35. doi: 10.1158/2767-9764.CRC-25-0638 (PMC13172104; doi:10.1158/2767-9764.CRC-25-0638)
Supplement: Table S3 — Representativeness of study participants with advanced CRC. [file crc-25-0638_table_s3_suppst3.docx]

**Table S3.** Representativeness of study participants with advanced CRC.

| **Patient populations** | Pooled manuscript population from two Phase Ia/Ib studies | General population: advanced, refractory gastrointestinal cancers |
| --- | --- | --- |
| **Considerations related to:** |  |  |
| Sex | Of the 122 patients assessed, 57% were male and 43% were female. | Overall, CRC incidence is ~32% higher in men than in women overall (40.5 vs. 30.7 cases per 100,000), although differences in incidence between sexes can vary by the patient age group and location of the tumor.^1,2^ |
| Age | Median ages were 61.0 and 54.5 years in the two studies. | According to the US National Cancer Institute, the median age of patients at diagnosis of CRC is ~67 years for colorectal cancer.^3^ This aligns with the American Cancer Society age-distribution data, which shows that 56% of new CRC cases occur at age ≥65 years, 32% at age 50–64 years, and 13% under age 50 years.^2^ |
| Race/ethnicity | Of the 122 patients enrolled across the two studies, 70.5% were White, 18% were Asian, 3.3% were Black or African-American, and 8.2% had other or missing information on race. | CRC incidence is highest in American Indian/Alaska Native and Black populations and is lowest in Asian/Pacific Islander populations. Multiple peer-reviewed analyses confirm this pattern, noting that Black and American Indian populations consistently experience the highest incidence.^1,4^ |
| Geography | These two studies recruited patients from sites in the following countries: Belgium, China, France, Germany, Japan, South Korea, Spain, and USA. | In the USA, overall CRC incidence is 35.3 per 100,000 people and is the leading cause of cancer death in adults aged <50 years.^1^ Outside the USA in 2020, CRC incidence rates were highest in Australia/ New Zealand and European regions (40.6 per 100 000, males) and lowest in several African regions and Southern Asia (4.4 per 100 000, females).^5^ |
| Overall representativeness of this study | In terms of sex and age or participants, the current analysis reflects the CRC incidence data shown in the wider literature. The overall racial composition of the study populations is reflective of that of the general populations of the countries that patients were recruited from and may be further influenced by the requirement to meet study inclusion criteria. Therefore, the racial composition of the current analysis does not accurately reflect the real-world racial subgroups that are predisposed to develop CRC. | |

CRC, colorectal cancer

1. Siegel RL, *et al*. Colorectal cancer statistics. CA: A Cancer Journal for Clinicians. 2026;76: https://doi.org/10.3322/caac.70067.
2. American Cancer Society. Colorectal Cancer Facts & Figures 2023-2025. Atlanta: American Cancer Society; 2023. Available online <https://www.cancer.org/content/dam/cancer-org/research/cancer-facts-and-statistics/colorectal-cancer-facts-and-figures/colorectal-cancer-facts-and-figures-2023.pdf> accessed March 2026.
3. National Cancer Institute. Cancer Causes and Prevention. <https://seer.cancer.gov/statfacts/html/all.html>, accessed Mach 2026.
4. Pankratz VS, et al. Cancer Incidence Trends Across Regions of the United States From 2001 to 2020– A United States Cancer Statistics Analysis. Cancer Control. 2024; 10732748241300653.
5. Morgan E, et al. Global burden of colorectal cancer in 2020 and 2040: incidence and mortality estimates from GLOBOCAN Gut 2023;72:338-344.
