# Supplementary material for: BI 905711, a TRAILR2/CDH17 Bispecific Antibody, Alone or with Chemotherapy for Patients with Advanced Gastrointestinal Cancers: Phase I Study Findings
Source: Cancer Res Commun. 2026 May 14;6(5):1123–35. doi: 10.1158/2767-9764.CRC-25-0638 (PMC13172104; doi:10.1158/2767-9764.CRC-25-0638)
Supplement: Table S4 — Summary of the most frequently reported adverse events by CTCAE preferred term for BI 905711 as monotherapy (>10% of patients) or combination therapy (≥25% of patients). [file crc-25-0638_table_s4_suppst4.docx]

**Table S4.** Summary of the most frequently reported adverse events by CTCAE preferred term for BI 905711 as monotherapy (>10% of patients) or combination therapy (≥25% of patients)*.*

| **AE by preferred term, *n* (%)** | **BI 905711 monotherapy (NCT04137289)  Phase Ia/Ib  (*n* = 110)^a^** | **BI 905711 combination therapy (NCT05087992)  Phase Ia/Ib  (*n* = 12)^b^** |
| --- | --- | --- |
| Any AE | 103 (93.6) | 12 (100.0) |
| Nausea | 28 (25.5) | 8 (66.7) |
| Abdominal pain | 24 (21.8) | 5 (41.7)^c^ |
| Anemia | 22 (20.0) | 3 (25.0) |
| Vomiting | 19 (17.3) | 4 (33.3) |
| AST increased | 18 (16.4) | 5 (41.7) |
| Fatigue | 17 (15.5) | 3 (25.0) |
| Asthenia | 16 (14.5) | - |
| Constipation | 16 (14.5) | 2 (16.7) |
| Pyrexia | 15 (13.6) | 4 (33.3) |
| Decreased appetite | 15 (13.6) | 11 (91.7) |
| Dyspnea | 15 (13.6) | - |
| Diarrhea | 14 (12.7) | 8 (66.7) |
| Cough | 13 (11.8) | - |
| Neutrophil count decreased | - | 7 (58.3) |
| White blood cell count decreased | - | 6 (50.0) |
| ALT increased |  | 5 (41.7) |
| Hypokalemia | - | 3 (25.0) |
| Proteinuria | - | 3 (25.0) |

Abbreviations: AE, adverse event; AST, aspartate aminotransferase; ALT, alanine aminotransferase; CTCAE, Common Terminology Criteria for Adverse Events; DILI, drug-induced liver injury.

^a^One patient had a grade 4 event of general physical health deterioration (unrelated to treatment); grade 5 events were reported by 13 patients (11.8%), all unrelated to treatment, with the majority (*n* = 8) being due to malignant neoplasm progression.

^b^Grade 3 AEs were diarrhea (three patients), neutrophil count decreased, white blood cell count decreased (two patients each), and one event each of stomatitis, decreased appetite, fatigue, COVID-19, gastroenteritis (*E. coli* infection), anemia, leukopenia, neutropenia, thrombocytopenia, DILI, and liver disorder. grade 4 AEs were reported in two patients (blood bilirubin increased and leukopenia).

^c^Includes preferred terms of ‘abdominal pain’ and abdominal pain upper’.
