# Supplementary material for: BI 905711, a TRAILR2/CDH17 Bispecific Antibody, Alone or with Chemotherapy for Patients with Advanced Gastrointestinal Cancers: Phase I Study Findings
Source: Cancer Res Commun. 2026 May 14;6(5):1123–35. doi: 10.1158/2767-9764.CRC-25-0638 (PMC13172104; doi:10.1158/2767-9764.CRC-25-0638)
Supplement: Table S5 — Individual pharmacokinetic parameters of cycle 3 based on ADA status in monotherapy study. [file crc-25-0638_table_s5_suppst5.docx]

**Table S5.** Individual pharmacokinetic parameters of cycle 3 based on ADA status in monotherapy study.

| **Study phase** | **Dose group** | **Number of PK and ADA evaluable patients** | **Individual C_max_ (ng/mL)** | | **Individual T_max_**  **(h)** | | **Individual AUC_0-336_**  **(h*ng/mL)** | |
| --- | --- | --- | --- | --- | --- | --- | --- | --- |
|  |  |  | **ADA-positive** | **ADA-negative** | **ADA-positive** | **ADA-negative** | **ADA-positive** | **ADA-negative** |
| 1a | 0.02 mg/kg Q2W | 1 | 336 | - | 0.483 | - | - | - |
|  | 0.06 mg/kg Q2W | 1 | - | 497 | - | 0.517 | - | 24500 |
|  | 0.2 mg/kg Q2W | 3 | 2290  3150 | 2380 | 0.450  0.500 | 0.550 | 87400  91200 | 141000 |
|  | 0.6 mg/kg Q2W | 6 | 6620  6000  10400 | 7160  5890  6920 | 0.517  0.500  0.467 | 0.550  0.467  0.550 | 173000  456000  421000 | 399000  391000  409000 |
|  | 1.2 mg/kg Q2W | 4 | - | 10200  5840  16300  11700 | - | 0.483  0.650  0.483  0.483 | - | 745000  541000  900000  728000 |
|  | 2.4 mg/kg Q2W | 6 | 23900  41400  36100 | 22500  33300  39500 | 0.500  0.467  0.450 | 6.90  0.483  0.550 | 771000  -  3810000 | -  2130000  2490000 |
|  | 3.6 mg/kg Q2W | 8 | 71300 | 33000  44000  41100  51300  52500  41500  56700 | 0.483 | 0.500  0.500  0.617  0.467  0.467  7.25  0.500 | 2170000 | 1870000  3720000  4230000  3950000  3470000  -  5880000 |
|  | 4.8 mg/kg Q2W | 6 | 44300 | 35000  48800  85900  42300  59200 | 0.567 | 23.8  0.483  0.533  0.483  0.983 | 3080000 | -  3120000  9990000  2210000  4740000 |
| 1b | 0.6 mg/kg Q2W | 10 | 6790  8580  5510  8560  2000 | 10100  4960  7110  7260  6990 | 0.583  0.633  0.500  0.417  0.500 | 0.500  0.583  0.483  7.00  0.550 | 446000  248000  210000  497000  - | 4020000  -  163000  378000  604000 |
|  | 1.2 mg/kg Q2W | 11 | 9340  21200  12500 | 11800  8950  8870  14500  29500  11000  14900  18000 | 0.483  0.567  0.483 | 6.80  6.90  6.78  0.533  0.533  0.483  0.483  0.467 | 388000  133000  1070000 | 1050000  468000  508000  240000  1050000  715000  708000  1190000 |
|  | 2.4 mg/kg Q2W | 12 | 23900  19700  21500  20700  20700 | 27900  28500  17200  21400  33800  18400  28700 | 0.433  0.500  0.583  0.750  0.567 | 0.500  0.500  7.00  0.500  0.500  1.08  0.483 | 1450000  1710000  1940000  511000  1570000 | 1710000  1680000  -  789000  2410000  951000  3270000 |
|  | 0.6 mg/kg QW | 12 | 4790  4850  5290  5450  8090 | 5850  3420  4930  4570  7960  5800  8920 | 6.78  6.72  6.87  1.00  0.483 | 0.600  1.00  0.483  6.88  0.500  0.483  0.500 | -  392000  475000  426000  - | 234000  185000  262000  245000  467000  436000  692000 |

Please note that number of PK and ADA evaluable patients of cycle 3 may differ from total reported number of PK evaluable patients and ADA evaluable patients.
Abbreviations: ADA, anti-drug antibody; AUC, area under the curve; C_max_, maximum concentration; PK, pharmacokinetics; Q2W, every 2 weeks; T_max_, time to maximum concentration.
