# Supplementary material for: BI 905711, a TRAILR2/CDH17 Bispecific Antibody, Alone or with Chemotherapy for Patients with Advanced Gastrointestinal Cancers: Phase I Study Findings
Source: Cancer Res Commun. 2026 May 14;6(5):1123–35. doi: 10.1158/2767-9764.CRC-25-0638 (PMC13172104; doi:10.1158/2767-9764.CRC-25-0638)
Supplement: Table S6 — Individual pharmacokinetic parameters of cycle 3 based on ADA status in combination study. [file crc-25-0638_table_s6_suppst6.docx]

**Table S6.** Individual pharmacokinetic parameters of cycle 3 based on ADA status in combination study.

| **Dose group** | **Number of PK/ADA evaluable patients** | **Individual  C_max_ (ng/mL)** | | **Individual  T_max_ (h)** | | **Individual  AUC_0-336_ (h*ng/mL)** | |
| --- | --- | --- | --- | --- | --- | --- | --- |
|  |  | **ADA-positive** | **ADA-negative** | **ADA-positive** | **ADA-negative** | **ADA-positive** | **ADA-negative** |
| 0.6 mg/kg Q2W | 9 | 7670  6380  4880  11800  6800 | 5830  6130  5590  6730 | 0.500  0.550  0.500  0.517  0.533 | 0.483  23.8  0.517  0.500 | 780000  515000  119000  666000  311000 | 311000  -  167000  454000 |
| 1.2 mg/kg Q2W | 3 | 7580  8780  12000 | - | 0.583  0.500  0.433 | - | 428000  755000  558000 | - |

Please note that number of PK and ADA evaluable patients of cycle 3 may differ from total reported number of PK evaluable patients and ADA evaluable patients.
Abbreviations: ADA, anti-drug antibody; AUC, area under the curve; C_max_, maximum concentration; PK, pharmacokinetics; Q2W, every 2 weeks; T_max_, time to maximum concentration.
