# Supplementary material for: BI 905711, a TRAILR2/CDH17 Bispecific Antibody, Alone or with Chemotherapy for Patients with Advanced Gastrointestinal Cancers: Phase I Study Findings
Source: Cancer Res Commun. 2026 May 14;6(5):1123–35. doi: 10.1158/2767-9764.CRC-25-0638 (PMC13172104; doi:10.1158/2767-9764.CRC-25-0638)
Supplement: Figure S1 — Molecular structure and mechanism of action of BI 905711. [file crc-25-0638_figure_s1_suppsf1.docx]

**Figure S1.** Molecular structure and mechanism of action of BI 905711.

**Molecular structure**


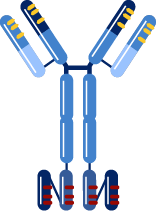


CDH17 binding domain

TRAILR2 binding domain
scFv (lexatumumab)

**BI 905711**

IgG1 LALA knock-out

**Mechanism of action**

**Cancer**

**CDH17-positive**

**Apoptosis**


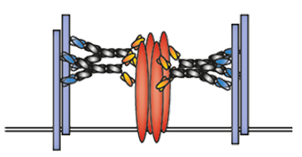

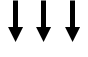


**Normal liver**

No apoptosis

**CDH17-negative**


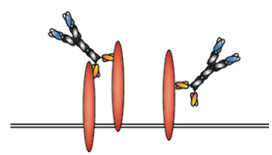

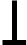


BI 905711, a tetravalent, IgG1, bispecific antibody cross-links TRAILR2 (red ovals) with CDH17 (blue boxes) on the surface of tumor cells to induce CDH17-dependent TRAILR2 oligomerization leading to CDH17-dependent apoptosis (left panel). In the absence of CDH17 (right panel) BI 905711-dependent apoptosis does not occur.

CDH17, cadherin 17; IgG1, immunoglobulin G1; LALA, Leu234Ala and Leu235Ala mutations; scFV, single-chain variable fragment; TRAILR2, tumor necrosis factor–related apoptosis-inducing ligand receptor 2.
