# Supplementary material for: BI 905711, a TRAILR2/CDH17 Bispecific Antibody, Alone or with Chemotherapy for Patients with Advanced Gastrointestinal Cancers: Phase I Study Findings
Source: Cancer Res Commun. 2026 May 14;6(5):1123–35. doi: 10.1158/2767-9764.CRC-25-0638 (PMC13172104; doi:10.1158/2767-9764.CRC-25-0638)
Supplement: Figure S2 — Study design of BI 905711 as A) monotherapy (NCT04137289) or B) combination therapy (NCT05087992). [file crc-25-0638_figure_s2_suppsf2.docx]

**Figure S2.** Study design of BI 905711 as A) monotherapy (NCT04137289) or B) combination therapy (NCT05087992).

**A)**


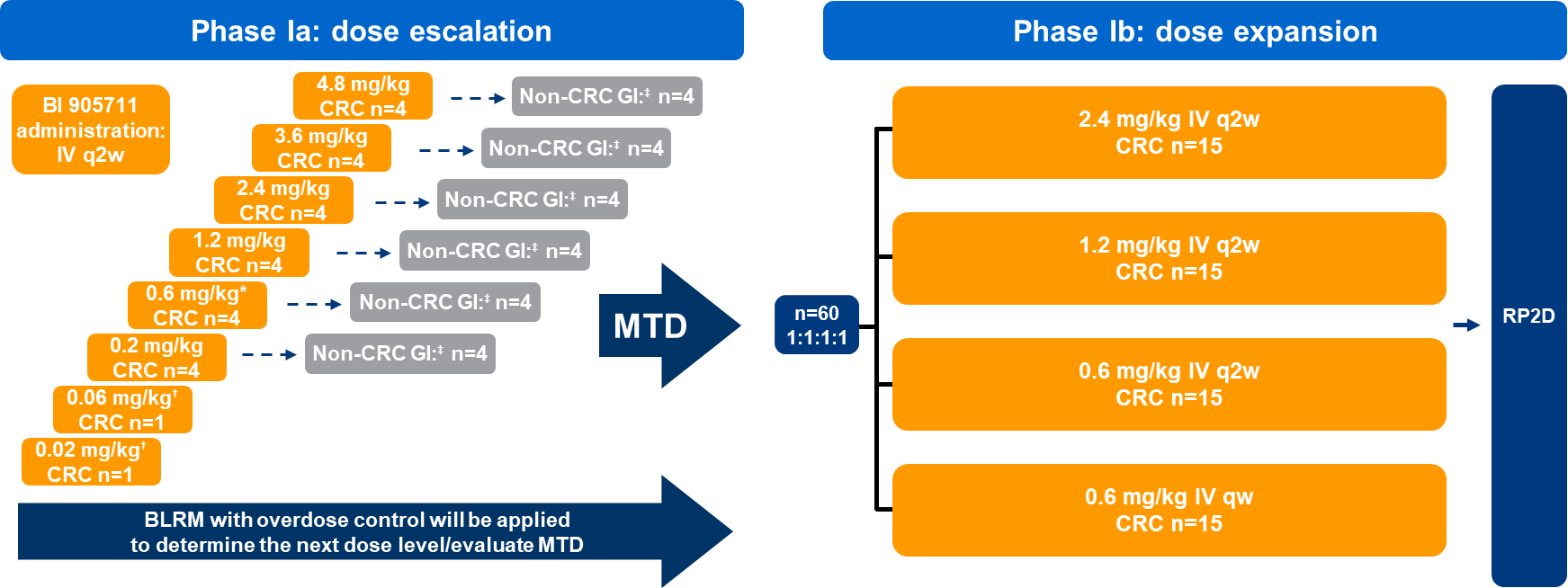


*****Predicted therapeutic dose; **^†^**If patients do not experience a DLT within the first two cycles, enrollment into a higher dose level can occur; if a DLT occurs, the number of patients will be increased to four per dose level; **^‡^**Patients with non-CRC GI cancer will be recruited as ‘back-filled’ cohorts at one level below the current dose being investigated in the CRC cohort.

BLRM, Bayesian Logistic Regression Model; CRC, colorectal cancer; DLT, dose-limiting toxicity; GI, gastrointestinal; IV, intravenous; MTD, maximum tolerated dose; qw, weekly regimen (3 weeks on, 1 week off); q2w, once every two weeks; RP2D, recommended Phase II dose.

**B)**


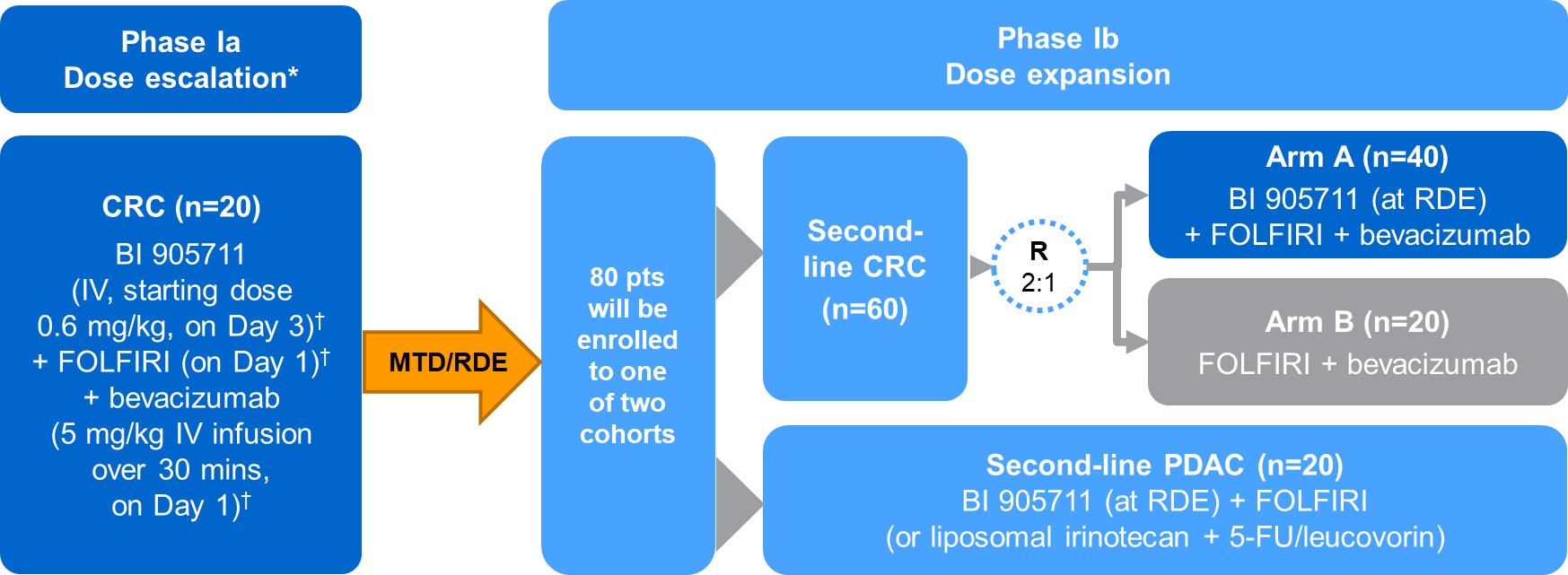


*Dose escalation will be guided by a Bayesian Logistic Regression Model with overdose control based on DLTs during the MTD evaluation period (first two 14-day cycles); ^†^Each cycle is 14 days.

5-FU, fluorouracil. CRC, colorectal cancer; DLT, dose-limiting toxicities; FOLFIRI, irinotecan, leucovorin, 5-FU; IV, intravenous; MTD, maximum tolerated dose; PDAC, pancreatic ductal adenocarcinoma; pts, patients; R, randomization; RDE, recommended dose for expansion.
