# Supplementary material for: BI 905711, a TRAILR2/CDH17 Bispecific Antibody, Alone or with Chemotherapy for Patients with Advanced Gastrointestinal Cancers: Phase I Study Findings
Source: Cancer Res Commun. 2026 May 14;6(5):1123–35. doi: 10.1158/2767-9764.CRC-25-0638 (PMC13172104; doi:10.1158/2767-9764.CRC-25-0638)
Supplement: Figure S3 — Maximum percentage change from baseline in the sum of target lesion diameters in study NCT04137289. [file crc-25-0638_figure_s3_suppsf3.docx]

**Figure S3.** Maximum percentage change from baseline in the sum of target lesion diameters in study NCT04137289.


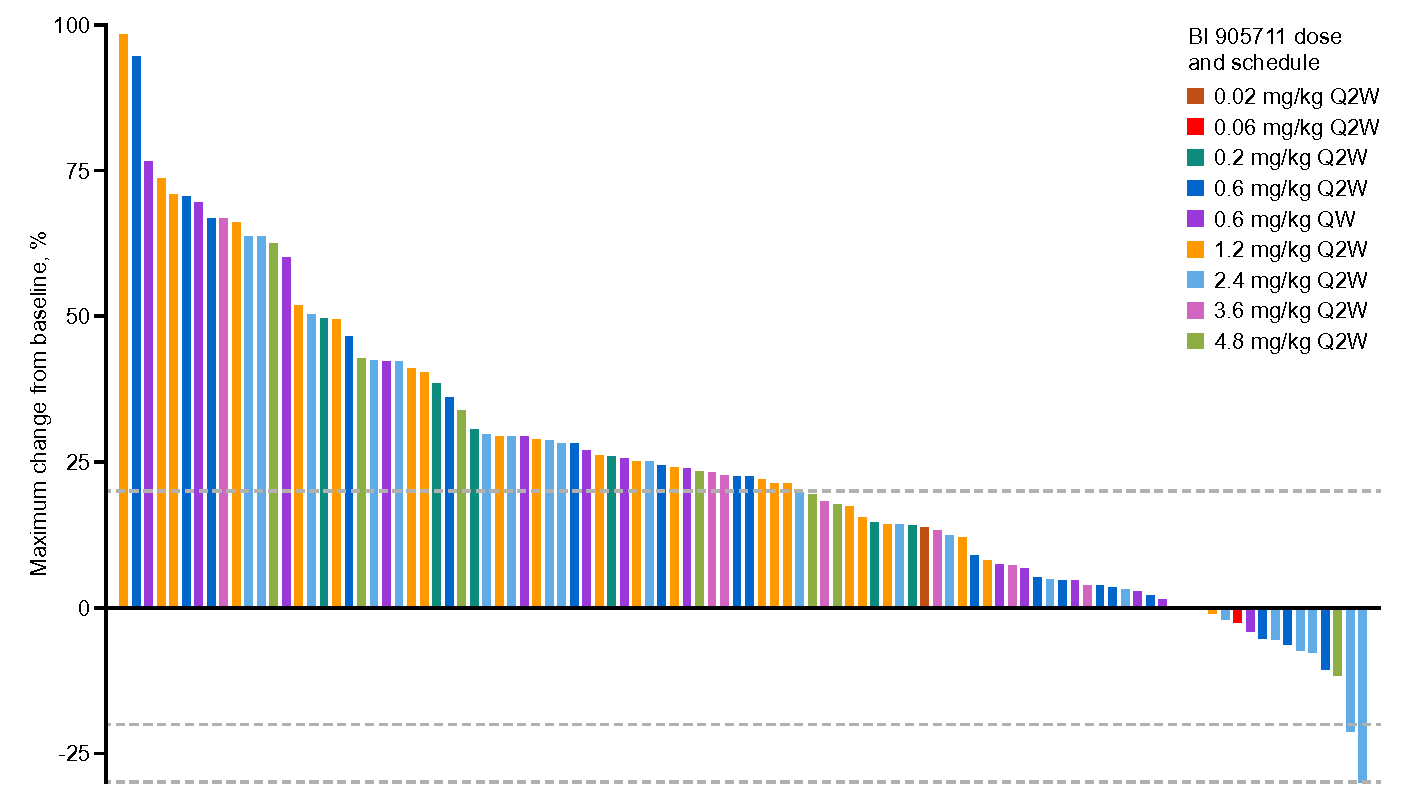


QW, once weekly; Q2W, once every 2 weeks.
